# Human fetal heart specific coexpression network involves congenital heart disease/defect candidate genes

**DOI:** 10.1038/srep46760

**Published:** 2017-04-24

**Authors:** Bo Wang, Guoling You, Qihua Fu

**Affiliations:** 1 Department of Laboratory Medicine, Shanghai Children’s Medical Center, Shanghai Jiaotong University School of Medicine, Shanghai, China

## Abstract

Heart development is a complex process requiring dynamic transcriptional regulation. Disturbance of this process will lead to severe developmental defects such as congenital heart disease/defect (CHD). CHD is a group of complex disorder with high genetic heterogeneity, common pathways associated with CHD remains largely unknown. In the manuscript, we focused on the tissue specific genes in human fetal heart samples to explore such pathways. We used the RNA microarray dataset of human fetal tissues from ENCODE project to identify genes with heart tissue specific expression. A transcriptional network was constructed for these genes based on the Pearson correlation coefficients of their expression levels. Function, selective constraints and disease associations of these genes were then examined. Our analysis identified a network consisted of 316 genes with human fetal heart specific expression. The network was highly co-regulated and showed evolutionary conserved tissue expression pattern in tetrapod. Genes in this network are enriched in CHD specific genes and disease mutations. Using the transcriptomic data, we discovered a highly concerted gene network that might reflect a common pathway associated with the etiology of CHD. Such analysis should be helpful for disease associated gene identification in clinical studies.

The heart is the first organ to form during animal embryonic development. A normal heart is crafted from a batch of progenitor cells that would underwent migrating, expanding and diversifying^1^. During early heart development, two masses of mesoderm migrate to the ventral midline and converge to form a primitive tubular heart. Then it transforms from a simple linear tubular structure to a four-chambered heart. Although the timing of the heart formation events varies, this process is generally similar in human and animal models^2^. Such developmental processes require dynamic and coordinate transcriptional processes orchestrated by cardiac transcription factors. Several pivotal signaling pathways (i.e., Bmp, Wnt, Notch, FGF, Hippo) involving in cardiac differentiation and specification by affecting critical cardiac transcription factors have been identified in cell lines and model organisms^3^,^4^,^5^,^6^,^7^,^8^.

CHD is the most common birth defect worldwide and encompasses a wide range of heart malformation that involves defects in septal, valve and outflow tract. Although rapid improvement in diagnosis, intervention, surgery has dramatically increased the survival of neonatal with CHD^9^,^10^,^11^, patients with moderate and severe CHD could not be anatomically corrected. Adults who have underwent corrective procedures also need to be monitored for risk of arrhythmias, endocarditis, and heart failure. Along with the complex cellular and molecular mechanism underlying heart development, most CHD cases has multifactorial etiologies, including genetic and environmental factors. The characterized causes of CHD could be summarized as the following: 1. chromosomal and single gene mutation disorders (~8%); 2. environmental teratogens (~2%); 3. complex multifactorial etiology (~90%). Non-inherited environmental factors such as pregestational diabetes, pollakiuria, rubeola, influenza febrile illnesses, alcohol, cigarette and teratogenic chemical agents have been revealed as important risk factors^12^. CHD could occur as autosomal dominant, autosomal recessive, X-linked or polygenic^13^. Although a collection of scattered evidences had established the mutational basis for some syndromic and non-syndromic CHD cases^13^,^14^,^15^,^16^, the genetic architecture of CHD still remains incompletely understood.

Given the heterogeneity of CHD, it would be valuable to identify common molecular pathways associated with this developmental disorder. Since highly coexpression of genes functioning in common processes is a widespread phenomenon in eukaryotes^17^, transcriptomic data should be especially suitable for detecting such common mechanisms. Tissue specific gene expression plays critical roles in human development. A full understanding of these genes could help revealing the molecular mechanisms underlying organ development and associated diseases^18^. In fact, it has been proved that the expression pattern of some tissue specific genes could be indicators for many complex diseases, such as insulin signaling genes in diabetes and stroma-tumor interaction genes in cancer.

Here, we identified 316 human fetal heart tissue specific genes using the ENCODE RNA microarray data. A highly co-regulated transcriptional network of these genes was constructed based on their expression level across the human fetal tissues. Disease mutation genes and CHD candidate genes were shown to be overrepresented in the network. Our results indicated that the co-regulation of tissue specific expression genes in human fetal hearts should have important sense to heart development and CHD etiology.

## Results

### Coexpression network of human fetal heart specific gene expression

**Figure 1:**
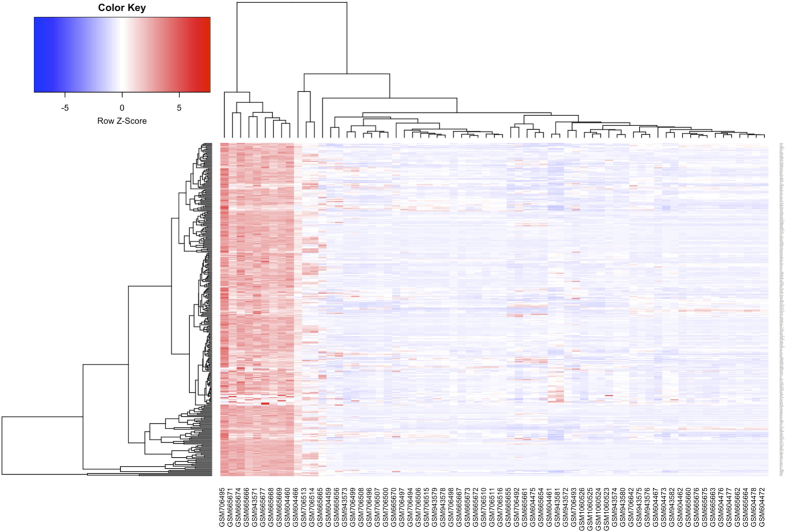
Heatmap of expression of 316 human fetal heart specific genes.

Totally, 1581 genes with TSI range from 0.144 to 0.932 show max expression in fetal heart samples. 316 tissue specific genes (TSI, 0.621–0.932) were selected as human fetal heart specific genes (Fig. 1, Supplementary Table S2) based on the calculation of TSI (Equation 1) score (top 20%).

**Figure 2:**
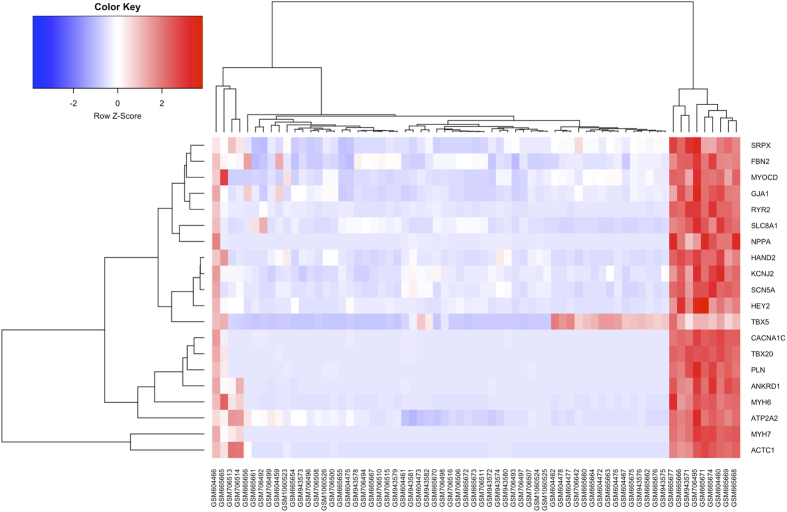
Heatmap of expression of 20 known CHD genes in human fetal heart specific genes.

We used the expression values of the 316 genes in heart samples to calculate the Pearson correlation coefficient matrix. It’s notable that CHD candidate genes are enriched (*p* < 0.001) in the tissue specific genes (20 genes: *PLN, NPPA, ANKRD1, MYH6, MYH7, ACTC1, CACNA1C, TBX20, HEY2, SLC8A1, RYR2, MYOCD, GJA1, ATP2A2, FBN2, SRPX, SCN5A, TBX5, HAND2, KCNJ2*) (Fig. 2).

**Figure 3:**
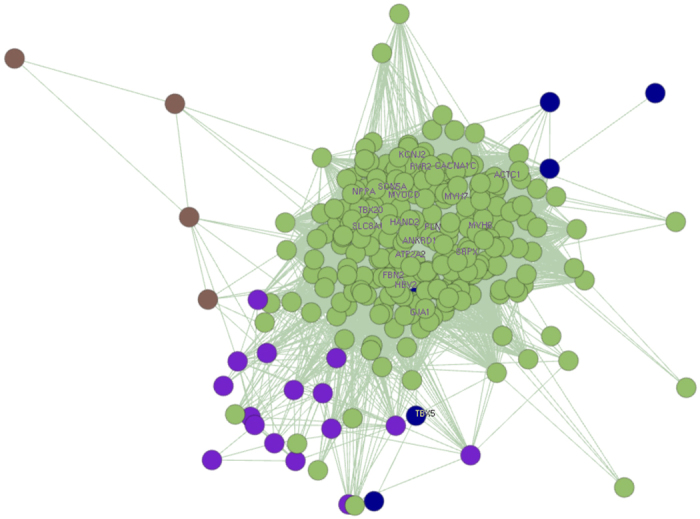
Co-expression network for 316 human fetal heart specific genes.

We then constructed the coexpression network for the 316 tissue specific genes with the Pearson correlation coefficients greater than 0.8 as edges. 4 clusters were detected in the network, with the largest one encompasses most (90%) of all genes (Fig. 3). Correlation coefficients of the 316 genes were shown in Figure S1. Finally, we also used the STRING database to validate the molecular interaction among the 316 genes, a network that has significantly more interactions than expected was detected. Interestingly, the network is centered by several CHD candidate genes (Supplementary Figure S2, S3).

### Functional enrichment analysis of human fetal heart specific genes

**Figure 4:**
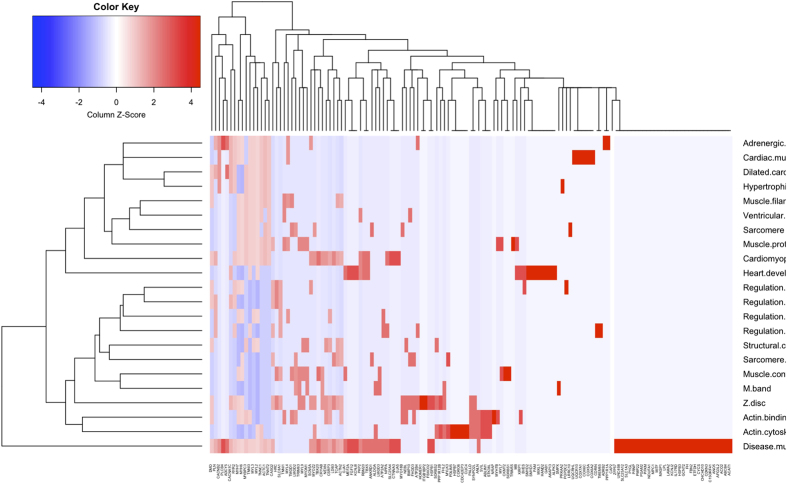
Heatmap of relationship of human fetal heart specific genes with significantly enriched functional items.

The 316 human fetal heart tissues specific genes are enriched in GO terms associated with processes such as regulation of muscle contraction, muscle organ development, and heart development. Genes in the coexpression network may be critical to orchestrating disease specific pathways such as Adrenergic signaling in cardiomyocyte, cardiac muscle contraction, and dilated cardiomyopathy. Additionally, these genes are also significantly enriched in disease mutation (*p* = 3.2e-8) (Table 1, Fig. 4).

### Relaxed selective constraints in human fetal heart specific genes

**Figure 5:**
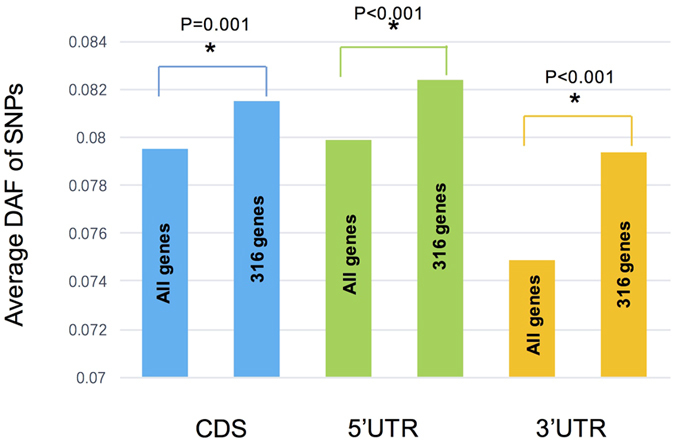
Comparison of the average derived allele frequency for the 316 human fetal heart specific genes and all protein coding genes.

Since disease mutations tend to occur in the human fetal heart specific genes, we expect that these genes are prone to harbor more nuclear acid substitution in the evolutionary process. Consistent with this assumption, single nucleotide polymorphisms (SNPs) in these genes segregating in African population have significantly lower derived allele frequencies (Fig. 5).

### Tissue expression of Human fetal heart specific genes in 11 tetrapod

**Figure 6:**
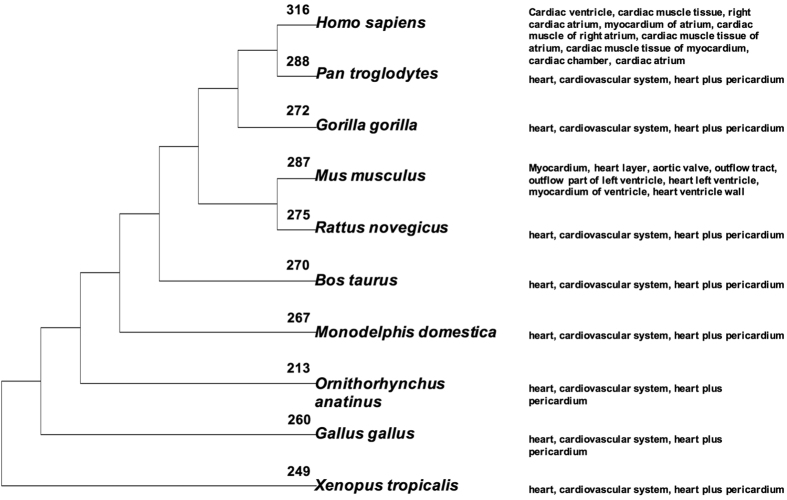
Tissue specification of the 316 gene orthologs in 10 tetrapod. The numbers of genes that have corresponding orthologs for each species are labeled on the branches.

Bgee database integrated together RNA-seq, microarray and *in situ* hybridization data from tens of animal species. We used the Bgee to assess tissue expression of human fetal heart tissue specific gene orthologs across 10 tetrapod species. The numbers of genes that have ortholog(s) for each species are: chimpanzee (288), gorilla (272), mouse (287), rat (275), cow (270), opossum (267), platypus (213), chicken (260), frog (249). The genes showed enriched expression in human heart related structures. Comparison of the expression patterns indicate that the tissue specificities are similar across tetrapod (Fig. 6).

## Discussion

Genes function as members of molecular pathways, and these pathways crosstalk with each other to form a complex regulatory network. To understand how the molecular mechanism is disrupted for a specific disease, the modules normally working in healthy tissues or cells should also be revealed firstly. Genes with co-regulation patterns should be of similar functional significance. When it refers to the developmental issues, gene expression profiles should be especially important. A number of genes and genetic networks contribute to the spatial and temporal specification that is necessary for normal embryological heart formation^14^,^19^,^20^,^21^. In our study, we focused on the genes that show specific expression in human fetal heart tissues. The high correlated expression pattern of the genes indicate that they are co-regulated during heart development. It’s noteworthy that 20 CHD candidate genes identified in clinical studies are important component in the coexpression network. These genes may interact with other nodes in the network not only at the transcriptional level but also at the protein level. The tissue expression pattern of the network should be generally conserved at least in tetrapod. Further investigation is needed to reveal the stability of the network across the whole stage of human heart development and model animals.

CHD originated from early development, thus many cases accompany chromosomal syndromes such as Trisomy 21, Trisomy 18, Trisomy 13, Turner’s syndrome, DiGeorge syndrome, Williams-Beuren syndrome, Alagille syndrome, Char syndrome, and Tetrasomy 22q. The early origin of CHD etiology could also explain why the tissue specific genes we identified are enriched in pathways such as neurological disease.

Non-syndromic or isolated CHDs are believed to arise from point mutations in genes that could affect heart development through haploinsufficiency or reduction in the dosage of encoded proteins. The known CHD genes play roles in transcriptional regulation, signal transduction, or encoding cardiac structural proteins^13^. Recently, progresses have been achieved for elucidating CHD genetic etiology^22^,^23^,^24^,^25^. However, since the genetics of CHDs is highly heterogeneous, the identification of CHD associated gene mutations are inefficient. The network we discovered in human fetal heart specific genes should represent a candidate common pathway close related to the development of CHD. In fact, detecting common pathways for complex diseases from gene expression data of normal tissues have been proved to be viable^26^. Our results indicated that such analysis should be valuable for priority selection of genes in clinical genetics study. Additionally, based on the 1000genome data, DAF of SNPs in the human fetal heart specific genes segregating in African population is significantly lower. From an evolutionary viewpoint, this could be attributed to relaxed selective constraint of purifying selection. The result suggested that screening of pathogenic mutations for these genes in clinical samples should also be meaningful.

In summary, we constructed a highly co-regulated transcriptional network of genes from tissue specific genes in human fetal heart tissues. Comparison of tissue expression among 11 tetrapoda species indicate that the network should be evolutionarily conserved. The network is enriched in CHD candidate genes and disease mutations. Such a transcriptional network might represent a common pathway associated with heart development and CHD, experimental validation of the gene network is needed. The results also indicate that gene expression data should be helpful in clinical studies for pathogenic mutation identification.

## Methods

### Datasets

The Exon microarray (Human Exon 1.0 ST) gene expression data (quantile normalized with PM-GCBG background correction and PLIER summarized) of human fetal tissues were downloaded from human ENCODE project^27^. The detailed information for tissue samples and NCBI Gene Expression Omnibus (GEO) accession numbers has been listed in Supplementary Table S1. The normalization between arrays were achieved by using the R package limma with the method scaling the arrays to have the same median. The NetAffx transcript cluster annotation file (release 36) were downloaded for annotation of the protein coding genes. For the transcripts that assigned for the same gene, the smallest one that could cover the coding sequence (CDS) was kept. We used the mean expression level if more than one sample could be used for the same tissue type at each time point. All analysis was completed using custom scripts in python or R.

### Human heart specific expression gene identification

A previously proposed tissue specificity index^28^ (TSI):


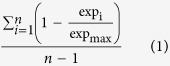


was used to calculate the tissue specificity for each gene, where n is the number of evaluated tissues, exp_i_ is the expression value in tissue I, and exp_max_ is the maximum expression level across all the tissues. The index varies between 0 (housekeeping genes without tissue specificity) and 1 (tissue-restricted genes with extreme tissue specificity). Firstly, we identified the genes that show exp_max_ in either of the 10 heart tissues. Secondly, we computed the TSI for the them and selected the top 20% as human fetal heart specific genes according to the TSI value.

### Network and Gene ontology analysis

Pearson correlation coefficient matrix was computed for the human fetal heart specific genes we identified. The network was constructed for the gene set with high co-regulation relationship (correlation coefficient ≥0.80) as edges. We visualized the network with BioLayout *Express*^3D^ 29 and detected highly inter connected gene clusters with the MCL (Markov Cluster) algorithm^30^. STRING v10.0^31^ (http://www.string-db.org) were also used to analyze the putative functional association networks for these genes. Gene ontology (GO) analysis was performed with the database for annotation, visualization, and integrated discovery (DAVID) software^32^,^33^ (https://david.ncifcrf.gov). The list of CHD candidate genes was acquired from DisGeNET database^34^ (http://www.disgenet.org).

### Selective constraint on DNA sequences

Genetic variant data were downloaded from the 1000 genome project^35^. The average derived allele frequency (DAF) for the African population was computed for the human fetal heart specific gene set and all gene set respectively. Since the parametric statistics could not be used due to non-normal distributions, we derived 95% confidence intervals from 500 bootstrap resampling replicates for DAF comparisons.

### Expression of homologous across tetrapoda species

We aquired the ortholog information of the human fetal heart specific gene for 9 tetrapoda species: chimpanzee (*Pan paniscus*), gorilla (*Gorilla gorilla*), mouse (*Mus musculus*), rat (*Rattus novegicus*), cattle (*Bos taurus*), opossum (*Monodelphis domestica*), platypus (*Ornithorhynchus anatinus*), chicken (*Gallus gallus*), xenopus (*Xenopus tropicalis*) from OMA (Orthologous MAtrix) (http://omabrowser.org) and made comparisons of the tissue expression of the orthologs across these species using the Bgee gene expression database^36^,^37^ (http://www.bgee.org).

## Additional Information

**How to cite this article:** Wang, B. *et al*. Human fetal heart specific coexpression network involves congenital heart disease/defect candidate genes. *Sci. Rep.* **7**, 46760; doi: 10.1038/srep46760 (2017).

**Publisher's note:** Springer Nature remains neutral with regard to jurisdictional claims in published maps and institutional affiliations.

## Supplementary Material

Supplementary Information

## Figures and Tables

**Table 1:** Significantly functional enriched items.

Enrichment terms	Gene counts of heart specific genes	p-value	Benjamini
Cardiomyopathy	28	2.9e-29	8.5e-27
Z disc	32	3.7e-29	9.3e-27
Cardiac muscle contraction	18	3.7e-19	5.5e-16
Muscle protein	19	1.1e-18	1.7e-16
Muscle filament sliding	15	8.2e-16	5.8e-13
Cardiac muscle contraction	18	8.4e-15	1.5e-12
Sarcomere	14	2.1e-14	2.6e-12
Ventricular cardiac muscle tissue morphogenesis	11	4.6e-12	2.3e-9
Actin binding	26	5.4e-12	2.7e-9
Heart development	21	4.5e-11	1.7e-8
Structural constituent of muscle	12	5.2e-11	1.3e-8
Adrenergic signaling in cardiomyocytes	19	7.5e-11	6.8e-9
Actin-binding	23	2.5e-10	2.4e-8
Muscle contraction	16	3.8e-10	1.1e-7
Regulation of the force of heart contraction	9	4.1e-10	1.0e-7
Sarcomere organization	10	7.9e-10	1.7e-7
Regulation of heart rate	10	2.9e-9	5.3e-7
Actin cytoskeleton	19	1.6e-8	1.3e-6
Disease mutation	75	2.3e-8	1.7e-6
Dilated cardiomyopathy	13	2.5e-8	1.5e-6
