# Supplementary material for: Human fetal heart specific coexpression network involves congenital heart disease/defect candidate genes
Source: Sci Rep. 2017 Apr 24;7:46760. doi: 10.1038/srep46760 (PMC5402266; doi:10.1038/srep46760)
Supplement: Supplementary Information [file srep46760-s1.pdf]

Human fetal heart specific coexpression network involves congenital heart disease/defect candidate genes

Bo Wang[1], Guoling You[1], Qihua Fu[1*]

1. Department of Laboratory Medicine, Shanghai Children’ s Medical Center, Shanghai Jiaotong University School of Medicine, Shanghai, China

*corresponding author

Fax: 86-21-58756923

Email: qihuafu@126.com

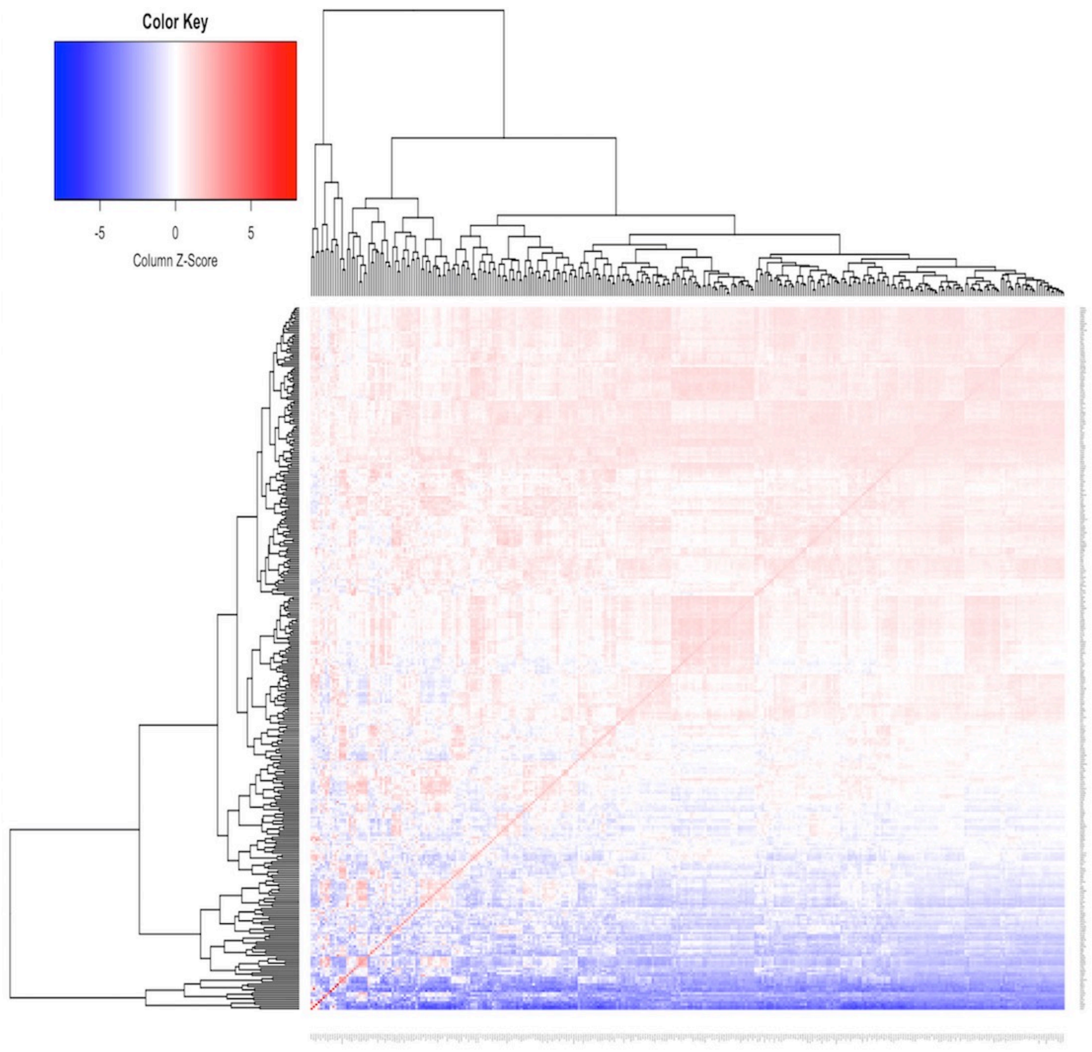

Figure S1 Heatmap of correlation coefficients of the 316 human fetal heart specific genes.

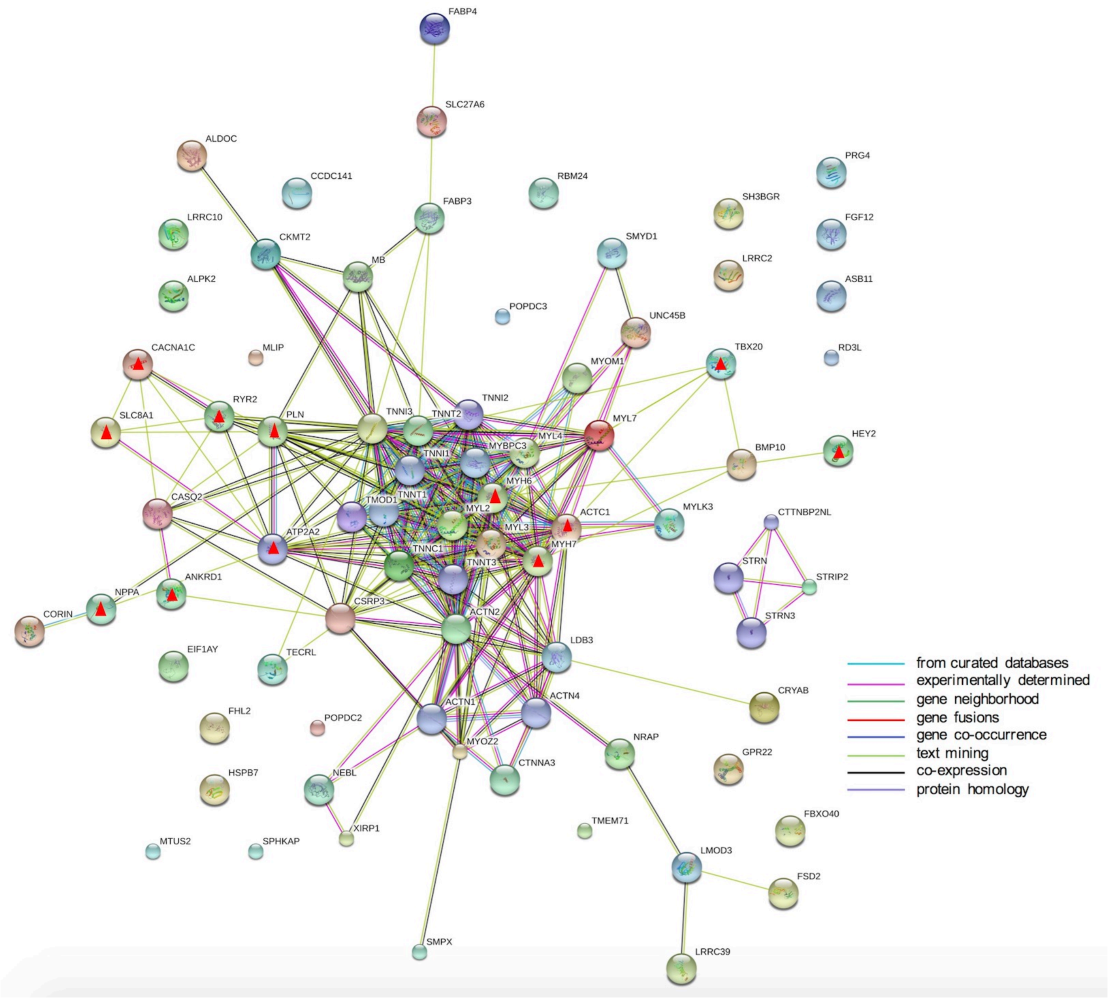

Figure S2 STRING Interaction of the 316 human fetal heart specific genes.

Table S1 Sample Information.

GEO_ID	Stage	Tissue
GSM604460	96 day	heart
GSM604466	101 day	heart
GSM665666	117 day	heart
GSM665668	96 day	heart
GSM665669	103 day	heart
GSM665671	105 day	heart
GSM665674	147 day	heart
GSM665677	110 day	heart
GSM706495	110 day	heart
GSM943571	105 day	heart
GSM604477	85 day	lung
GSM604476	67 day	lung
GSM604472	108 day	lung
GSM604462	120 day	lung
GSM604467	101 day	lung
GSM604478	96 day	lung
GSM604473	103 day	lung
GSM665660	108 day	lung
GSM665662	112 day	lung
GSM665663	82 day	lung
GSM1060525	110 day	small intestine
GSM706496	115 day	small intestine
GSM706499	105 day	small intestine
GSM943573	87 day	small intestine
GSM943574	91 day	small intestine
GSM943580	107 day	small intestine
GSM706508	108 day	small intestine
GSM604475	117 day	brain
GSM706492	122 day	brain
GSM665654	85 day	brain
GSM665655	96 day	brain
GSM665661	112 day	brain
GSM1060523	103 day	large intestine
GSM1060524	105 day	large intestine
GSM1060526	110 day	large intestine
GSM706500	107 day	large intestine
GSM706507	108 day	large intestine
GSM665665	117 day	left lung
GSM665676	110 day	left lung
GSM943576	91 day	left lung

**GSM943581**	107 day	left lung
**GSM706642**	113 day	left lung
**GSM665673**	147 day	left kidney
**GSM706494**	110 day	left kidney
**GSM706498**	115 day	left kidney
**GSM943578**	107 day	left kidney
**GSM665667**	117 day	right kidney
**GSM665672**	147 day	right kidney
**GSM706497**	115 day	right kidney
**GSM943579**	107 day	right kidney
**GSM665664**	117 day	right lung
**GSM665675**	110 day	right lung
**GSM943575**	91 day	right lung
**GSM943582**	107 day	right lung
**GSM706506**	108 day	renal cortex interstitium
**GSM706511**	108 day	renal cortex interstitium
**GSM706516**	113 day	renal cortex interstitium
**GSM604459**	96 day	adrenal gland
**GSM665656**	85 day	adrenal gland
**GSM604461**	120 day	kidney
**GSM665670**	105 day	kidney
**GSM706510**	108 day	renal pelvis
**GSM706515**	113 day	renal pelvis
**GSM706493**	147 day	thymus
**GSM943572**	105 day	thymus
**GSM706514**	115 day	muscle of arm
**GSM706513**	115 day	muscle of leg

Table S2 List of the human fetal heart specific genes.

Symbol	ENSEMBL ID	Notes
**BMP10**	ENSG00000163217	
**SMPX**	ENSG00000091482	
**TMEM71**	ENSG00000165071	
**PLN**	ENSG00000198523	CHD
**TECRL**	ENSG00000205678	
**NRAP**	ENSG00000197893	
**FGF12**	ENSG00000114279	
**CSRP3**	ENSG00000129170	
**MB**	ENSG00000198125	
**ALPK2**	ENSG00000198796	
**GPR22**	ENSG00000172209	
**NPPA**	ENSG00000175206	CHD
**ANKRD1**	ENSG00000148677	CHD
**FABP4**	ENSG00000170323	
**FBXO40**	ENSG00000163833	
**MYL2**	ENSG00000111245	
**RD3L**	ENSG00000227729	
**MYH7**	ENSG00000092054	CHD
**MYLK3**	ENSG00000140795	
**MYL3**	ENSG00000160808	
**FHL2**	ENSG00000115641	
**POPDC3**	ENSG00000132429	
**CTNNA3**	ENSG00000183230	
**MYH6**	ENSG00000197616	CHD
**POPDC2**	ENSG00000121577	
**TNNI3**	ENSG00000129991	
**EIF1AY**	ENSG00000198692	
**TNNT2**	ENSG00000118194	
**NEBL**	ENSG00000078114	
**ACTC1**	ENSG00000159251	CHD
**MPC1L**	ENSG00000238205	
**LMOD3**	ENSG00000163380	
**TNNC1**	ENSG00000114854	
**MYL4**	ENSG00000198336	
**MYOM1**	ENSG00000101605	
**LRRC10**	ENSG00000198812	
**SLC27A6**	ENSG00000113396	
**CACNA1C**	ENSG00000151067	CHD
**LRRC39**	ENSG00000122477	
**SH3BGR**	ENSG00000185437	

**TBX20**	ENSG00000164532	CHD
**CASQ2**	ENSG00000118729	
**MYOZ2**	ENSG00000172399	
**HEY2**	ENSG00000135547	CHD
**ACTN2**	ENSG00000077522	
**RBM24**	ENSG00000112183	
**SMYD1**	ENSG00000115593	
**FABP3**	ENSG00000121769	
**CCDC141**	ENSG00000163492	
**MYL7**	ENSG00000106631	
**MYBPC3**	ENSG00000134571	
**LRRC2**	ENSG00000163827	
**MLIP**	ENSG00000146147	
**ASB11**	ENSG00000165192	
**HSPB7**	ENSG00000173641	
**SLC8A1**	ENSG00000183023	CHD
**CRYAB**	ENSG00000109846	
**SPHKAP**	ENSG00000153820	
**CKMT2**	ENSG00000131730	
**LDB3**	ENSG00000122367	
**CORIN**	ENSG00000145244	
**PRG4**	ENSG00000116690	
**UNC45B**	ENSG00000141161	
**XIRP1**	ENSG00000168334	
**FSD2**	ENSG00000186628	
**RYR2**	ENSG00000198626	CHD
**STRIP2**	ENSG00000128578	
**ALDOC**	ENSG00000109107	
**MTUS2**	ENSG00000132938	
**TMOD1**	ENSG00000136842	
**COX7A1**	ENSG00000161281	
**FILIP1**	ENSG00000118407	
**GPRIN3**	ENSG00000185477	
**KLHL38**	ENSG00000175946	
**LMOD2**	ENSG00000170807	
**PKP2**	ENSG00000057294	
**MYOCD**	ENSG00000141052	CHD
**ITGB1BP2**	ENSG00000147166	
**PFKP**	ENSG00000067057	
**NEXN**	ENSG00000162614	
**GK2**	ENSG00000196475	
**AKAP6**	ENSG00000151320	
**HACD1**	ENSG00000165996	

**FHOD3**	ENSG00000134775	
**FITM1**	ENSG00000139914	
**PLCXD3**	ENSG00000182836	
**TMEM65**	ENSG00000164983	
**B4GALNT3**	ENSG00000139044	
**FAM129A**	ENSG00000135842	
**CACNB2**	ENSG00000165995	
**SORBS2**	ENSG00000154556	
**GABRA4**	ENSG00000109158	
**NAV1**	ENSG00000134369	
**PPP1R12B**	ENSG00000077157	
**GJA1**	ENSG00000152661	CHD
**CYP2J2**	ENSG00000134716	
**PDK4**	ENSG00000004799	
**TNNI1**	ENSG00000159173	
**MDH1**	ENSG00000014641	
**PAM**	ENSG00000145730	
**PXDNL**	ENSG00000147485	
**ADPRHL1**	ENSG00000153531	
**ACAT1**	ENSG00000075239	
**MLF1**	ENSG00000178053	
**PDLIM5**	ENSG00000163110	
**HHATL**	ENSG00000010282	
**DPY19L2**	ENSG00000177990	
**ANO5**	ENSG00000171714	
**NDUFA8**	ENSG00000119421	
**RBM20**	ENSG00000203867	
**C10orf71**	ENSG00000177354	
**USP28**	ENSG00000048028	
**SLC25A4**	ENSG00000151729	
**ASB2**	ENSG00000100628	
**PYGB**	ENSG00000100994	
**RBPMS2**	ENSG00000166831	
**BVES**	ENSG00000112276	
**HRC**	ENSG00000130528	
**SLC4A3**	ENSG00000114923	
**LIN9**	ENSG00000183814	
**FBXO32**	ENSG00000156804	
**LPL**	ENSG00000175445	
**GABRE**	ENSG00000102287	
**TRIM63**	ENSG00000158022	
**CDH2**	ENSG00000170558	
**LAMA2**	ENSG00000196569	

**GPNMB**	ENSG00000136235	
**PALLD**	ENSG00000129116	
**MYO18B**	ENSG00000133454	
**SLC1A3**	ENSG00000079215	
**ZFP57**	ENSG00000204644	
**TCAP**	ENSG00000173991	
**CHRM2**	ENSG00000181072	
**OR1N1**	ENSG00000171505	
**FGF18**	ENSG00000156427	
**MYH7B**	ENSG00000078814	
**NDUFA1**	ENSG00000125356	
**RCSD1**	ENSG00000198771	
**COX5A**	ENSG00000178741	
**ADAM23**	ENSG00000114948	
**ATP2A2**	ENSG00000174437	CHD
**BNIP3**	ENSG00000176171	
**DMD**	ENSG00000198947	
**PRKAA2**	ENSG00000162409	
**PGAM2**	ENSG00000164708	
**ANLN**	ENSG00000011426	
**ABCA9**	ENSG00000154258	
**CDC42EP3**	ENSG00000163171	
**XPO4**	ENSG00000132953	
**LBH**	ENSG00000213626	
**PDK1**	ENSG00000152256	
**FBN2**	ENSG00000138829	CHD
**PRND**	ENSG00000171864	
**USP2**	ENSG00000036672	
**SLC41A1**	ENSG00000133065	
**MYL9**	ENSG00000101335	
**RHOQ**	ENSG00000119729	
**BCO2**	ENSG00000197580	
**RIMKLA**	ENSG00000177181	
**PRSS46**	ENSG00000261603	
**SRPX**	ENSG00000101955	CHD
**DCUN1D2**	ENSG00000150401	
**SCN5A**	ENSG00000183873	CHD
**NMRK2**	ENSG00000077009	
**PRSS35**	ENSG00000146250	
**CT47B1**	ENSG00000236446	
**PAIP2B**	ENSG00000124374	
**PLEKHA7**	ENSG00000166689	
**HECW2**	ENSG00000138411	

**PBK**	ENSG00000168078	
**FBXL7**	ENSG00000183580	
**USMG5**	ENSG00000173915	
**CLGN**	ENSG00000153132	
**STK38L**	ENSG00000211455	
**TBX5**	ENSG00000089225	CHD
**FH**	ENSG00000091483	
**AHSG**	ENSG00000145192	
**OIT3**	ENSG00000138315	
**ADRB2**	ENSG00000169252	
**CA14**	ENSG00000118298	
**CAP2**	ENSG00000112186	
**MITF**	ENSG00000187098	
**HSPB1**	ENSG00000106211	
**PPARGC1A**	ENSG00000109819	
**CISD1**	ENSG00000122873	
**PLCB4**	ENSG00000101333	
**CNOT11**	ENSG00000158435	
**ABHD18**	ENSG00000164074	
**SVIL**	ENSG00000197321	
**HAND2**	ENSG00000164107	CHD
**GMPR**	ENSG00000137198	
**PPP1R1A**	ENSG00000135447	
**FAM177B**	ENSG00000197520	
**AFAP1L1**	ENSG00000157510	
**MPC1**	ENSG00000060762	
**CDKN3**	ENSG00000100526	
**ATP2B4**	ENSG00000058668	
**MFGE8**	ENSG00000140545	
**PYGL**	ENSG00000100504	
**DKK4**	ENSG00000104371	
**GOT1**	ENSG00000120053	
**KIDINS220**	ENSG00000134313	
**SYTL5**	ENSG00000147041	
**ABLIM1**	ENSG00000099204	
**LAPTM4B**	ENSG00000104341	
**SFRP1**	ENSG00000104332	
**STRN**	ENSG00000115808	
**ALPK3**	ENSG00000136383	
**ARHGAP31**	ENSG00000031081	
**RPL3L**	ENSG00000140986	
**PCDH7**	ENSG00000169851	
**CORO6**	ENSG00000167549	

**OR51B6**	ENSG00000176239	
**BZW2**	ENSG00000136261	
**PFKM**	ENSG00000152556	
**LMO7**	ENSG00000136153	
**MEF2A**	ENSG00000068305	
**PRSS45**	ENSG00000188086	
**SORBS1**	ENSG00000095637	
**FASTKD1**	ENSG00000138399	
**KCNJ2**	ENSG00000123700	CHD
**RCAN2**	ENSG00000172348	
**OR51I2**	ENSG00000187918	
**SAMD4A**	ENSG00000020577	
**ETFDH**	ENSG00000171503	
**UGP2**	ENSG00000169764	
**KCNJ8**	ENSG00000121361	
**GJA5**	ENSG00000265107	
**GPAT3**	ENSG00000138678	
**MIXL1**	ENSG00000185155	
**TRIM55**	ENSG00000147573	
**PRNP**	ENSG00000171867	
**RGS5**	ENSG00000232995	
**IL1RAPL1**	ENSG00000169306	
**PODN**	ENSG00000174348	
**UQCR10**	ENSG00000184076	
**CYCS**	ENSG00000172115	
**KBTBD12**	ENSG00000187715	
**FAM189A2**	ENSG00000135063	
**GPR52**	ENSG00000203737	
**KTN1**	ENSG00000126777	
**ISOC1**	ENSG00000066583	
**PPM1K**	ENSG00000163644	
**QKI**	ENSG00000112531	
**COX6A2**	ENSG00000156885	
**DECR1**	ENSG00000104325	
**GBAS**	ENSG00000146729	
**SMYD2**	ENSG00000143499	
**NDUFA6**	ENSG00000184983	
**HOMER1**	ENSG00000152413	
**MASP1**	ENSG00000127241	
**IL17RD**	ENSG00000144730	
**OMD**	ENSG00000127083	
**AKAP13**	ENSG00000170776	
**LRRFIP2**	ENSG00000093167	

**AFG3L2**	ENSG00000141385
**KIAA1456**	ENSG00000250305
**ACO2**	ENSG00000100412
**MGP**	ENSG00000111341
**SLC25A12**	ENSG00000115840
**LARGE**	ENSG00000133424
**UQCRHL**	ENSG00000233954
**USP13**	ENSG00000058056
**ZNF436**	ENSG00000125945
**CDH13**	ENSG00000140945
**TBC1D4**	ENSG00000136111
**ANKRD18A**	ENSG00000180071
**ABCB4**	ENSG00000005471
**GCNT2**	ENSG00000111846
**SOCS6**	ENSG00000170677
**CLIC4**	ENSG00000169504
**LRRC53**	ENSG00000162621
**RNF150**	ENSG00000170153
**TMEM159**	ENSG00000011638
**CAB39L**	ENSG00000102547
**COX6C**	ENSG00000164919
**ASB15**	ENSG00000146809
**LTBP1**	ENSG00000049323
**PPIC**	ENSG00000168938
**SYNPO2L**	ENSG00000166317
**CCDC179**	ENSG00000255359
**TACC2**	ENSG00000138162
**TGFB1I1**	ENSG00000140682
**ADCY5**	ENSG00000173175
**CKAP2**	ENSG00000136108
**LRR1**	ENSG00000165501
**MYBL1**	ENSG00000185697
**PPP1R1C**	ENSG00000150722
**PPP1R3C**	ENSG00000119938
**CHCHD10**	ENSG00000250479
**CRYBG3**	ENSG00000080200
**TSPAN9**	ENSG00000011105
**A4GALT**	ENSG00000128274
**IFNA16**	ENSG00000147885
**KLHL24**	ENSG00000114796
**PPP1R3B**	ENSG00000173281
**STARD4**	ENSG00000164211
**ADAL**	ENSG00000168803

**HSPB3**	ENSG00000169271
**PRSS42**	ENSG00000178055
**RRAD**	ENSG00000166592
**ASB14**	ENSG00000239388
**EGLN3**	ENSG00000129521
**MGST3**	ENSG00000143198
**NDUFB4**	ENSG00000065518
**C15orf41**	ENSG00000186073
**PLPP7**	ENSG00000160539
**PMP22**	ENSG00000109099
**ALDOA**	ENSG00000149925
**CPT1B**	ENSG00000205560
**DENND5A**	ENSG00000184014
**EIF1B**	ENSG00000114784
**LRRC14B**	ENSG00000185028
**PTP4A3**	ENSG00000184489
**GAB1**	ENSG00000109458
**SBSPON**	ENSG00000164764

Table S3 Coexpression network neighbors of the CHD genes identified in the study.

	Co-expressed genes
PLN	SMPX,TMEM71,TECRL,NRAP,FGF12,CSRP3,MB,ALPK2,GPR22,NPPA,ANKRD1,FBXO40,MYL2,RD3L,MYH7,MYLK3,MYL3,FHL2,POPDC3,CTNNA3,MYH6,POPDC2,TNNI3,TNNT2,NEBL,ACTC1,MPC1L,LMOD3,TNNC1,MYL4,MYOM1,LRRC10,SLC27A6,CACNA1C,LRRC39,SH3BGR,TBX20,CASQ2,MYOZ2,HEY2,ACTN2,RBM24,SMYD1,FABP3,CCDC141,MYL7,MYBPC3,LRRC2,MLIP,ASB11,HSPB7,SLC8A1,CRYAB,SPHKAP,CKMT2,LDB3,CORIN,PRG4,UNC45B,XIRP1,FSD2,RYR2,STRIP2,ALDOC,MTUS2,TMOD1,COX7A1,FILIP1,GPRIN3,KLHL38,LMOD2,PKP2,MYOCD,ITGB1BP2,PFKP,NEXN,GK2,AKAP6,HACD1,FHOD3,FITM1,PLCXD3,TMEM65,B4GALNT3,FAM129A,CACNB2,SORBS2,GABRA4,NAV1,PPP1R12B,GJA1,CYP2J2,TNNI1,MDH1,PAM,ADPRHL1,ACAT1,MLF1,PDLIM5,HHATL,DPY19L2,ANO5,RBM20,C10orf71,USP28,SLC25A4,ASB2,PYGB,RBPMS2,BVES,HRC,SLC4A3,LIN9,FBXO32,TRIM63,CDH2,LAMA2,GPNMB,PALLD,MYO18B,TCAP,CHRM2,FGF18,MYH7B,NDUFA1,RCSD1,COX5A,ADAM23,ATP2A2,DMD,PRKAA2,PGAM2,ANLN,ABCA9,CDC42EP3,XPO4,LBH,PDK1,FBN2,USP2,MYL9,RHOQ,BCO2,RIMKLA,SRPX,DCUN1D2,SCN5A,NMRK2,CT47B1,PAIP2B,PLEKHA7,HECW2,FBXL7,CLGN,STK38L,OIT3,ADRB2,CA14,CAP2,MITF,HSPB1,PPARGC1A,PLCB4,CNOT11,ABHD18,SVIL,HAND2,GMPR,PPP1R1A,AFAP1L1,MPC1,ATP2B4,MFGE8,GOT1,KIDINS220,SYTL5,ABLIM1,LAPTM4B,SFRP1,STRN,ALPK3,RPL3L,PCDH7,CORO6,OR51B6,BZW2,PFKM,MEF2A,PRSS45,SORBS1,FASTKD1,KCNJ2,RCAN2,SAMD4A,KCNJ8,GPAT3,TRIM55,RGS5,PODN,CYCS,KBTBD12,FAM189A2,ISOC1,QKI,COX6A2,DECR1,GBAS,SMYD2,AKAP13,LRRFIP2,AFG3L2,ACO2,SLC25A12,LARGE,USP13,ZNF436,ANKRD18A,ABCB4,CLIC4,LRRC53,RNF150,TMEM159,LTBP1,PPIC,TACC2,TGFB1I1,ADCY5,LRR1,MYBL1,CHCHD10,CRYBG3,TSPAN9,A4GALT,HSPB3,PRSS42,RRAD,MGST3,C15orf41,PLPP7,ALDOA,CPT1B,DENND5A,LRRC14B,PTP4A3,GAB1
NPPA	TMEM71,PLN,TECRL,NRAP,FGF12,CSRP3,MB,ALPK2,ANKRD1,FBXO40,MYL2,MYH7,MYLK3,MYL3,FHL2,POPDC3,CTNNA3,MYH6,POPDC2,TNNI3,TNNT2,NEBL,ACTC1,MPC1L,LMOD3,TNNC1,MYL4,MYOM1,LRRC10,SLC27A6,CACNA1C,SH3BGR,TBX20,CASQ2,MYOZ2,HEY2,ACTN2,RBM24,SMYD1,FABP3,CCDC141,MYL7,MYBPC3,LRRC2,MLIP,ASB11,HSPB7,SLC8A1,CRYAB,SPHKAP,CKMT2,LDB3,CORIN,PRG4,UNC45B,XIRP1,FSD2,RYR2,STRIP2,ALDOC,MTUS2,TMOD1,COX7A1,KLHL38,PKP2,MYOCD,ITGB1BP2,PFKP,NEXN,GK2,HACD1,FHOD3,FITM1,PLCXD3,TMEM65,B4GALNT3,FAM129A,CACNB2,SORBS2,GABRA4,NAV1,PPP1R12B,GJA1,TNNI1,MDH1,PAM,ADPRHL1,PDLIM5,HHATL,DPY19L2,ANO5,RBM20,C10orf71,USP28,SLC25A4,ASB2,PYGB,RBPMS2,BVES,HRC,SLC4A3,LIN9,FBXO32,TRIM63,LAMA2,PALLD,MYO18B,CHRM2,FGF18,MYH7B,ADAM23,ATP2A2,PGAM2,ANLN,CDC42EP3,XPO4,LBH,FBN2,USP2,MYL9,RHOQ,RIMKLA,SRPX,SCN5A,NMRK2,CT47B1,PAIP2B,PLEKHA7,FBXL7,CLGN,STK38L,ADRB2,CAP2,MITF,HSPB1,PPARGC1A,CNOT11,HAND2,PPP1R1A,MPC1,ATP2B4,MFGE8,GOT1,ABLIM1,STRN,ALPK3,CORO6,OR51B6,PFKM,MEF2A,PRSS45,KCNJ2,RCAN2,TRIM55,PODN,CYCS,KBTBD12,FAM189A2,COX6A2,GBAS,AKAP13,ACO2,SLC25A12,LARGE,ABCB4,CLIC4,LRRC53,TMEM159,LTBP1,TACC2,TGFB1I1,ADCY5,LRR1,MYBL1,CHCHD10,TSPAN9,A4GALT,HSPB3,PRSS42,RRAD,C15orf41,PLPP7,ALDOA,CPT1B,DENND5A,LRRC14B,PTP4A3
ANKR	SMPX,TMEM71,PLN,TECRL,NRAP,FGF12,CSRP3,MB,ALPK2,GPR22,NPPA,FBXO40,MYL2,RD3L,MYH7,MYLK3,MYL3,FHL2,POPDC3,CTNNA3,MYH6,POPDC2,TNNI3,TNNT2,NEBL,ACTC1,MPC1L,LMOD3,TNNC1,MYL4,MYOM1,LRRC10,SLC27A6,CACNA1C,LRRC39,SH3BGR,TBX20,CASQ2,MYOZ2,HEY2,ACTN2,RBM24,SMYD1,FABP3,CCDC141,MYL7,MYBPC3,LRRC2,MLIP,ASB11,HSPB7,SLC8A1,CRYAB,SPHKAP,CKMT2,LDB3,CORIN,UNC45B,XIRP1,FSD2,RYR2,STRIP2,ALDOC,MTUS2,TMOD1,COX7A1,FILIP1,GPRIN3,KLHL38,LMOD2,PKP2,MYOCD,ITGB1BP2,PFKP,NEXN,GK2,AKAP6,HACD1,FHOD3,FITM1,PLCXD3,TMEM65,B4GALNT3,FAM129A,CACNB2,SORBS2,GABRA4,NAV1,PPP1R12B,GJA1,CYP2J2,TNNI1,MDH1,PAM,ADPRHL1,ACAT1,MLF1,PDLIM5,HHATL,DPY19L2,ANO5,RBM20,C10orf71,USP28,SLC25A4,ASB2,PYGB,RBPMS2,BVES,HRC,SLC4A3,LIN9,FBXO32,TRIM63,LAMA2,GPNMB,PALLD,MYO18B,TCAP,CHRM2,FGF18,MYH7B,NDUFA1,RCSD1,COX5A,ADAM23,ATP2A2,DMD,PRKAA2,PGAM2,ANLN,ABCA9,CDC42EP3,XPO4,LBH,FBN2,USP2,MYL9,RHOQ,SRPX,DCUN1D2,SC

D1	N5A,NMRK2,CT47B1,PAIP2B,PLEKHA7,HECW2,FBXL7,CLGN,STK38L,OIT3,ADRB2,CA14,CAP2,MITF,HSPB1,PPARGC1A,CNOT11,ABHD18,SVIL,HAND2,GMPR,PPP1R1A,AFAP1L1,MPC1,ATP2B4,MFGE8,GOT1,ABLIM1,STRN,ALPK3,RPL3L,PCDH7,CORO6,OR51B6,BZW2,PFKM,MEF2A,PRSS45,SORBS1,KCNJ2,RCAN2,SAMD4A,KCNJ8,GPAT3,TRIM55,PODN,CYCS,KBTBD12,FAM189A2,QKI,COX6A2,GBAS,SMYD2,AKAP13,LRRFIP2,AFG3L2,ACO2,SLC25A12,LARGE,USP13,ZNF436,ANKRD18A,ABCB4,CLIC4,LRRC53,RNF150,TMEM159,LTBP1,PPIC,SYNPO2L,TACC2,TGFB1I1,ADCY5,LRR1,MYBL1,PPP1R3C,CHCHD10,CRYBG3,TSPAN9,A4GALT,HSPB3,PRSS42,RRAD,MGST3,C15orf41,PLPP7,ALDOA,CPT1B,DENND5A,LRRC14B,PTP4A3,GAB1
MYH6	SMPX,TMEM71,PLN,TECRL,NRAP,FGF12,CSRP3,MB,ALPK2,NPPA,ANKRD1,FBXO40,MYL2,MYH7,MYLK3,MYL3,FHL2,POPDC3,CTNNA3,POPDC2,TNNI3,TNNT2,NEBL,ACTC1,MPC1L,LMOD3,TNNC1,MYL4,MYOM1,LRRC10,SLC27A6,CACNA1C,LRRC39,SH3BGR,TBX20,CASQ2,MYOZ2,HEY2,ACTN2,RBM24,SMYD1,FABP3,CCDC141,MYL7,MYBPC3,LRRC2,MLIP,ASB11,HSPB7,SLC8A1,CRYAB,SPHKAP,CKMT2,LDB3,CORIN,UNC45B,XIRP1,FSD2,RYR2,STRIP2,ALDOC,MTUS2,TMOD1,COX7A1,FILIP1,KLHL38,LMOD2,PKP2,MYOCD,ITGB1BP2,PFKP,NEXN,GK2,HACD1,FHOD3,FITM1,PLCXD3,TMEM65,B4GALNT3,FAM129A,SORBS2,GABRA4,NAV1,PPP1R12B,GJA1,CYP2J2,TNNI1,MDH1,PAM,ADPRHL1,MLF1,PDLIM5,HHATL,DPY19L2,ANO5,RBM20,C10orf71,USP28,SLC25A4,ASB2,PYGB,RBPMS2,BVES,HRC,SLC4A3,LIN9,FBXO32,TRIM63,CDH2,LAMA2,GPNMB,PALLD,MYO18B,TCAP,CHRM2,FGF18,MYH7B,NDUFA1,RCSD1,COX5A,ADAM23,ATP2A2,DMD,PRKAA2,PGAM2,ANLN,CDC42EP3,XPO4,LBH,PDK1,FBN2,USP2,SLC41A1,MYL9,RHOQ,SRPX,SCN5A,NMRK2,CT47B1,PAIP2B,PLEKHA7,FBXL7,CLGN,STK38L,OIT3,ADRB2,CA14,CAP2,MITF,HSPB1,PPARGC1A,CNOT11,ABHD18,SVIL,HAND2,GMPR,PPP1R1A,AFAP1L1,MPC1,ATP2B4,MFGE8,PYGL,GOT1,SYTL5,ABLIM1,SFRP1,STRN,ALPK3,CORO6,OR51B6,BZW2,PFKM,MEF2A,KCNJ2,SAMD4A,TRIM55,PODN,CYCS,KBTBD12,FAM189A2,QKI,COX6A2,GBAS,SMYD2,AKAP13,LRRFIP2,ACO2,SLC25A12,LARGE,USP13,ANKRD18A,ABCB4,CLIC4,LRRC53,RNF150,TMEM159,LTBP1,PPIC,TACC2,ADCY5,LRR1,MYBL1,CHCHD10,TSPAN9,A4GALT,ADAL,PRSS42,RRAD,C15orf41,PLPP7,ALDOA,CPT1B,DENND5A,LRRC14B,PTP4A3
MYH7	SMPX,TMEM71,PLN,TECRL,NRAP,FGF12,CSRP3,MB,ALPK2,GPR22,NPPA,ANKRD1,FBXO40,MYL2,RD3L,MYLK3,MYL3,FHL2,POPDC3,CTNNA3,MYH6,POPDC2,TNNI3,TNNT2,NEBL,ACTC1,MPC1L,LMOD3,TNNC1,MYL4,MYOM1,LRRC10,SLC27A6,CACNA1C,LRRC39,SH3BGR,TBX20,CASQ2,MYOZ2,HEY2,ACTN2,RBM24,SMYD1,FABP3,CCDC141,MYL7,MYBPC3,LRRC2,MLIP,ASB11,HSPB7,SLC8A1,CRYAB,SPHKAP,CKMT2,LDB3,CORIN,PRG4,UNC45B,XIRP1,FSD2,RYR2,STRIP2,ALDOC,MTUS2,TMOD1,COX7A1,FILIP1,GPRIN3,KLHL38,LMOD2,PKP2,MYOCD,ITGB1BP2,PFKP,NEXN,GK2,AKAP6,HACD1,FHOD3,FITM1,PLCXD3,TMEM65,B4GALNT3,FAM129A,CACNB2,SORBS2,GABRA4,NAV1,PPP1R12B,GJA1,CYP2J2,TNNI1,MDH1,PAM,ADPRHL1,ACAT1,MLF1,PDLIM5,HHATL,DPY19L2,ANO5,RBM20,C10orf71,USP28,SLC25A4,ASB2,PYGB,RBPMS2,BVES,HRC,SLC4A3,LIN9,FBXO32,TRIM63,CDH2,LAMA2,GPNMB,PALLD,MYO18B,TCAP,CHRM2,FGF18,MYH7B,NDUFA1,RCSD1,COX5A,ADAM23,ATP2A2,DMD,PRKAA2,PGAM2,ANLN,ABCA9,CDC42EP3,XPO4,LBH,PDK1,FBN2,USP2,SLC41A1,MYL9,RHOQ,BCO2,RIMKLA,SRPX,DCUN1D2,SCN5A,NMRK2,CT47B1,PAIP2B,PLEKHA7,HECW2,FBXL7,CLGN,STK38L,OIT3,ADRB2,CA14,CAP2,MITF,HSPB1,PPARGC1A,PLCB4,CNOT11,ABHD18,SVIL,HAND2,GMPR,PPP1R1A,AFAP1L1,MPC1,ATP2B4,MFGE8,PYGL,GOT1,ABLIM1,SFRP1,STRN,ALPK3,RPL3L,PCDH7,CORO6,OR51B6,BZW2,PFKM,MEF2A,PRSS45,SORBS1,FASTKD1,KCNJ2,RCAN2,SAMD4A,KCNJ8,GPAT3,TRIM55,PODN,CYCS,KBTBD12,FAM189A2,ISOC1,QKI,COX6A2,GBAS,SMYD2,AKAP13,LRRFIP2,AFG3L2,ACO2,SLC25A12,LARGE,USP13,ZNF436,ANKRD18A,ABCB4,CLIC4,LRRC53,RNF150,TMEM159,LTBP1,PPIC,TACC2,TGFB1I1,ADCY5,LRR1,MYBL1,PPP1R3C,CHCHD10,CRYBG3,TSPAN9,A4GALT,HSPB3,PRSS42,RRAD,MGST3,C15orf41,PLPP7,ALDOA,CPT1B,DENND5A,LRRC14B,PTP4A3,GAB1
ACT	SMPX,PLN,TECRL,NRAP,FGF12,CSRP3,MB,ALPK2,NPPA,ANKRD1,FBXO40,MYL2,MYH7,MYLK3,MYL3,FHL2,POPDC3,CTNNA3,MYH6,POPDC2,TNNI3,TNNT2,NEBL,MPC1L,LMOD3,TNNC1,MYL4,MYOM1,LRRC10,SLC27A6,CACNA1C,LRRC39,SH3BGR,TBX20,CASQ2,MYOZ2,HEY2,ACTN2,RBM24,SMYD1,FABP3,CCDC141,MYL7,MYBPC3,LRRC2,MLIP,ASB11,HSPB7,SLC8A1,CRYAB,SPHKAP,CKMT2,LDB3,CORIN,UNC45B,XIRP1,FSD2,RYR2,STRIP2,ALDOC,MTUS2,TMOD1,COX7A1,FILIP1,KLHL38,LMOD2,PKP2,ITGB1BP2,PFKP,NEXN,GK2,AKAP6,HACD1,FHOD3,FITM1,PLCXD3,TMEM65,B4GALNT3,FAM129A,SORBS2,GABRA4,NAV1,PPP1R12B,GJA1,CYP2J2,TNNI1,MDH1,ADPRHL1,MLF1,PDLIM5,HHATL,ANO5,RBM20,C10orf71,USP28,SLC25A4,ASB2,PYGB,RBPMS2,BVES,HRC,SLC4A3,FBXO32,TRIM63,LAMA2,GPNMB,PALLD,MYO18B,TCAP,CHRM

C1	2,FGF18,MYH7B,NDUFA1,COX5A,ADAM23,ATP2A2,DMD,PRKAA2,PGAM2,ANLN,ABCA9,CDC42EP3,XPO4,LBH,FBN2,USP2,MYL9,RHOQ,SRPX,SCN5A,NMRK2,CT47B1,PAIP2B,PLEKHA7,FBXL7,CLGN,OIT3,ADRB2,CAP2,HSPB1,PPARGC1A,CNOT11,ABHD18,SVIL,HAND2,GMPR,PPP1R1A,AFAP1L1,MPC1,ATP2B4,MFGE8,PYGL,GOT1,ABLIM1,STRN,ALPK3,RPL3L,CORO6,OR51B6,BZW2,PFKM,MEF2A,SORBS1,KCNJ2,SAMD4A,KCNJ8,TRIM55,PODN,CYCS,KBTBD12,FAM189A2,QKI,COX6A2,GBAS,SMYD2,AKAP13,SLC25A12,USP13,ABCB4,LRRC53,RNF150,ASB15,LTBP1,SYNPO2L,CCDC179,TACC2,TGFB1I1,ADCY5,LRR1,MYBL1,CHCHD10,CRYBG3,TSPAN9,A4GALT,HSPB3,PRSS42,RRAD,MGST3,C15orf41,PLPP7,ALDOA,CPT1B,DENND5A,LRRC14B,PTP4A3
CACNA1C	SMPX,TMEM71,PLN,TECRL,NRAP,FGF12,CSRP3,MB,ALPK2,GPR22,NPPA,ANKRD1,FBXO40,MYL2,RD3L,MYH7,MYLK3,MYL3,FHL2,POPDC3,CTNNA3,MYH6,POPDC2,TNNI3,TNNT2,NEBL,ACTC1,MPC1L,LMOD3,TNNC1,MYL4,MYOM1,LRRC10,SLC27A6,LRRC39,SH3BGR,TBX20,CASQ2,MYOZ2,HEY2,ACTN2,RBM24,SMYD1,FABP3,CCDC141,MYL7,MYBPC3,LRRC2,MLIP,ASB11,HSPB7,SLC8A1,CRYAB,SPHKAP,CKMT2,LDB3,CORIN,PRG4,UNC45B,XIRP1,FSD2,RYR2,STRIP2,ALDOC,MTUS2,TMOD1,COX7A1,FILIP1,GPRIN3,KLHL38,LMOD2,PKP2,MYOCD,ITGB1BP2,PFKP,NEXN,GK2,AKAP6,HACD1,FHOD3,FITM1,PLCXD3,TMEM65,B4GALNT3,FAM129A,CACNB2,SORBS2,GABRA4,NAV1,PPP1R12B,GJA1,TNNI1,MDH1,PAM,ADPRHL1,ACAT1,MLF1,PDLIM5,HHATL,DPY19L2,ANO5,RBM20,C10orf71,USP28,SLC25A4,ASB2,PYGB,RBPMS2,BVES,HRC,SLC4A3,LIN9,FBXO32,LPL,TRIM63,CDH2,LAMA2,GPNMB,PALLD,MYO18B,TCAP,CHRM2,FGF18,MYH7B,NDUFA1,RCSD1,COX5A,ADAM23,ATP2A2,DMD,PRKAA2,PGAM2,ANLN,ABCA9,CDC42EP3,XPO4,LBH,FBN2,USP2,SLC41A1,MYL9,RHOQ,BCO2,RIMKLA,SRPX,DCUN1D2,SCN5A,NMRK2,CT47B1,PAIP2B,PLEKHA7,HECW2,FBXL7,CLGN,STK38L,OIT3,ADRB2,CA14,CAP2,MITF,HSPB1,PPARGC1A,PLCB4,CNOT11,ABHD18,SVIL,HAND2,GMPR,PPP1R1A,AFAP1L1,MPC1,ATP2B4,MFGE8,GOT1,ABLIM1,SFRP1,STRN,ALPK3,RPL3L,PCDH7,CORO6,OR51B6,BZW2,PFKM,MEF2A,PRSS45,SORBS1,FASTKD1,KCNJ2,RCAN2,SAMD4A,KCNJ8,TRIM55,PODN,CYCS,KBTBD12,FAM189A2,ISOC1,QKI,COX6A2,DECR1,GBAS,SMYD2,AKAP13,LRRFIP2,AFG3L2,ACO2,SLC25A12,LARGE,ZNF436,ANKRD18A,ABCB4,CLIC4,LRRC53,RNF150,TMEM159,LTBP1,PPIC,TACC2,TGFB1I1,ADCY5,LRR1,MYBL1,CHCHD10,CRYBG3,TSPAN9,A4GALT,HSPB3,PRSS42,RRAD,MGST3,C15orf41,PLPP7,ALDOA,CPT1B,DENND5A,LRRC14B,PTP4A3,GAB1
TBX20	SMPX,TMEM71,PLN,TECRL,NRAP,FGF12,CSRP3,MB,ALPK2,GPR22,NPPA,ANKRD1,FBXO40,MYL2,MYH7,MYLK3,MYL3,FHL2,POPDC3,CTNNA3,MYH6,POPDC2,TNNI3,TNNT2,NEBL,ACTC1,MPC1L,LMOD3,TNNC1,MYL4,MYOM1,LRRC10,SLC27A6,CACNA1C,LRRC39,SH3BGR,CASQ2,MYOZ2,HEY2,ACTN2,RBM24,SMYD1,FABP3,CCDC141,MYL7,MYBPC3,LRRC2,MLIP,ASB11,HSPB7,SLC8A1,CRYAB,SPHKAP,CKMT2,LDB3,CORIN,PRG4,UNC45B,XIRP1,FSD2,RYR2,STRIP2,ALDOC,MTUS2,TMOD1,COX7A1,FILIP1,GPRIN3,KLHL38,LMOD2,PKP2,MYOCD,ITGB1BP2,PFKP,NEXN,GK2,HACD1,FHOD3,FITM1,PLCXD3,TMEM65,B4GALNT3,FAM129A,CACNB2,SORBS2,GABRA4,NAV1,PPP1R12B,GJA1,TNNI1,MDH1,PAM,ADPRHL1,ACAT1,MLF1,PDLIM5,HHATL,DPY19L2,ANO5,RBM20,C10orf71,USP28,SLC25A4,ASB2,PYGB,RBPMS2,BVES,HRC,SLC4A3,LIN9,FBXO32,TRIM63,CDH2,LAMA2,GPNMB,PALLD,MYO18B,TCAP,CHRM2,FGF18,MYH7B,NDUFA1,RCSD1,COX5A,ADAM23,ATP2A2,DMD,PRKAA2,PGAM2,ANLN,ABCA9,CDC42EP3,XPO4,LBH,PDK1,FBN2,USP2,SLC41A1,MYL9,RHOQ,BCO2,RIMKLA,SRPX,DCUN1D2,SCN5A,NMRK2,PRSS35,CT47B1,PAIP2B,PLEKHA7,HECW2,FBXL7,CLGN,STK38L,OIT3,ADRB2,CA14,CAP2,MITF,HSPB1,PPARGC1A,PLCB4,CNOT11,ABHD18,SVIL,HAND2,GMPR,PPP1R1A,AFAP1L1,MPC1,ATP2B4,MFGE8,GOT1,SYTL5,ABLIM1,SFRP1,STRN,ALPK3,PCDH7,CORO6,OR51B6,BZW2,PFKM,MEF2A,PRSS45,SORBS1,FASTKD1,KCNJ2,RCAN2,SAMD4A,KCNJ8,TRIM55,PODN,CYCS,KBTBD12,FAM189A2,ISOC1,QKI,COX6A2,GBAS,SMYD2,AKAP13,LRRFIP2,AFG3L2,ACO2,SLC25A12,LARGE,USP13,ZNF436,ANKRD18A,ABCB4,CLIC4,LRRC53,RNF150,TMEM159,LTBP1,PPIC,TACC2,TGFB1I1,ADCY5,LRR1,MYBL1,CHCHD10,CRYBG3,TSPAN9,A4GALT,HSPB3,PRSS42,RRAD,MGST3,C15orf41,PLPP7,ALDOA,CPT1B,DENND5A,LRRC14B,PTP4A3,GAB1
HE	SMPX,TMEM71,PLN,TECRL,NRAP,FGF12,CSRP3,MB,ALPK2,GPR22,NPPA,ANKRD1,FBXO40,MYL2,RD3L,MYH7,MYLK3,MYL3,FHL2,POPDC3,CTNNA3,MYH6,POPDC2,TNNI3,TNNT2,NEBL,ACTC1,MPC1L,LMOD3,TNNC1,MYL4,MYOM1,LRRC10,SLC27A6,CACNA1C,LRRC39,SH3BGR,TBX20,CASQ2,MYOZ2,ACTN2,RBM24,SMYD1,FABP3,CCDC141,MYL7,MYBPC3,LRRC2,MLIP,ASB11,HSPB7,SLC8A1,CRYAB,SPHKAP,CKMT2,LDB3,CORIN,UNC45B,XIRP1,FSD2,RYR2,STRIP2,ALDOC,MTUS2,TMOD1,COX7A1,FILIP1,GPRIN3,KLHL38,LMOD2,PKP2,MYOCD,ITGB1BP2,PFKP,NEXN,GK2,AKAP6,HACD1,FHOD3,FIT

Y2	M1,PLCXD3,TMEM65,B4GALNT3,FAM129A,CACNB2,SORBS2,GABRA4,NAV1,PPP1R12B,GJA1,TNNI1,MDH1,ADPRHL1,ACAT1,MLF1,PDLIM5,HHATL,DPY19L2,ANO5,RBM20,C10orf71,USP28,SLC25A4,ASB2,PYGB,RBPMS2,BVES,HRC,SLC4A3,LIN9,FBXO32,TRIM63,LAMA2,PALLD,MYO18B,CHRM2,FGF18,MYH7B,COX5A,ADAM23,ATP2A2,DMD,PRKAA2,PGAM2,ANLN,CDC42EP3,XPO4,LBH,FBN2,USP2,MYL9,RHOQ,BCO2,SRPX,DCUN1D2,SCN5A,NMRK2,CT47B1,PAIP2B,PLEKHA7,FBXL7,CLGN,STK38L,ADRB2,CA14,CAP2,MITF,HSPB1,PPARGC1A,PLCB4,CNOT11,ABHD18,HAND2,GMPR,PPP1R1A,AFAP1L1,MPC1,MFGE8,GOT1,SYTL5,ABLIM1,STRN,ALPK3,PCDH7,CORO6,OR51B6,BZW2,PFKM,MEF2A,FASTKD1,KCNJ2,RCAN2,SAMD4A,KCNJ8,TRIM55,PODN,CYCS,KBTBD12,FAM189A2,QKI,COX6A2,GBAS,SMYD2,AKAP13,LRRFIP2,AFG3L2,ACO2,SLC25A12,LARGE,ZNF436,ABCB4,CLIC4,LRRC53,RNF150,TMEM159,LTBP1,PPIC,TACC2,TGFB1I1,ADCY5,LRR1,MYBL1,CHCHD10,CRYBG3,TSPAN9,A4GALT,HSPB3,PRSS42,RRAD,MGST3,C15orf41,PLPP7,CPT1B,DENND5A,LRRC14B,PTP4A3,GAB1
SLC8A1	TMEM71,PLN,TECRL,NRAP,FGF12,CSRP3,MB,ALPK2,GPR22,NPPA,ANKRD1,FBXO40,MYL2,MYH7,MYLK3,MYL3,FHL2,POPDC3,CTNNA3,MYH6,POPDC2,TNNI3,TNNT2,NEBL,ACTC1,MPC1L,LMOD3,TNNC1,MYL4,MYOM1,LRRC10,SLC27A6,CACNA1C,SH3BGR,TBX20,CASQ2,MYOZ2,HEY2,ACTN2,RBM24,SMYD1,FABP3,CCDC141,MYL7,MYBPC3,LRRC2,MLIP,ASB11,HSPB7,CRYAB,SPHKAP,CKMT2,LDB3,CORIN,UNC45B,XIRP1,FSD2,RYR2,STRIP2,ALDOC,MTUS2,TMOD1,COX7A1,FILIP1,GPRIN3,KLHL38,PKP2,ITGB1BP2,PFKP,NEXN,GK2,AKAP6,HACD1,FHOD3,FITM1,PLCXD3,TMEM65,B4GALNT3,FAM129A,CACNB2,SORBS2,GABRA4,NAV1,PPP1R12B,GJA1,TNNI1,MDH1,PAM,ADPRHL1,ACAT1,MLF1,PDLIM5,HHATL,DPY19L2,ANO5,RBM20,C10orf71,USP28,SLC25A4,ASB2,PYGB,RBPMS2,BVES,SLC4A3,LIN9,FBXO32,TRIM63,CDH2,LAMA2,GPNMB,PALLD,MYO18B,CHRM2,FGF18,MYH7B,NDUFA1,COX5A,ADAM23,ATP2A2,PRKAA2,PGAM2,ANLN,CDC42EP3,XPO4,LBH,FBN2,USP2,MYL9,RHOQ,RIMKLA,SRPX,DCUN1D2,SCN5A,NMRK2,CT47B1,PAIP2B,PLEKHA7,HECW2,FBXL7,CLGN,STK38L,ADRB2,CA14,CAP2,MITF,PPARGC1A,PLCB4,CNOT11,ABHD18,HAND2,PPP1R1A,MPC1,GOT1,KIDINS220,ABLIM1,SFRP1,STRN,ALPK3,PCDH7,CORO6,OR51B6,BZW2,PFKM,MEF2A,SORBS1,FASTKD1,KCNJ2,RCAN2,SAMD4A,TRIM55,PODN,CYCS,KBTBD12,PPM1K,QKI,COX6A2,GBAS,SMYD2,HOMER1,LRRFIP2,AFG3L2,KIAA1456,ACO2,SLC25A12,LARGE,ZNF436,ANKRD18A,ABCB4,CLIC4,LRRC53,RNF150,TMEM159,LTBP1,PPIC,TACC2,ADCY5,LRR1,MYBL1,CHCHD10,TSPAN9,A4GALT,PRSS42,RRAD,MGST3,C15orf41,PLPP7,CPT1B,DENND5A,LRRC14B,PTP4A3
RYR2	SMPX,TMEM71,PLN,TECRL,NRAP,FGF12,CSRP3,MB,ALPK2,GPR22,NPPA,ANKRD1,FBXO40,MYL2,RD3L,MYH7,MYLK3,MYL3,FHL2,POPDC3,CTNNA3,MYH6,POPDC2,TNNI3,TNNT2,NEBL,ACTC1,MPC1L,LMOD3,TNNC1,MYL4,MYOM1,LRRC10,SLC27A6,CACNA1C,LRRC39,SH3BGR,TBX20,CASQ2,MYOZ2,HEY2,ACTN2,RBM24,SMYD1,FABP3,CCDC141,MYL7,MYBPC3,LRRC2,MLIP,ASB11,HSPB7,SLC8A1,CRYAB,SPHKAP,CKMT2,LDB3,CORIN,PRG4,UNC45B,XIRP1,FSD2,STRIP2,ALDOC,MTUS2,TMOD1,COX7A1,FILIP1,GPRIN3,KLHL38,LMOD2,PKP2,MYOCD,ITGB1BP2,PFKP,NEXN,GK2,AKAP6,HACD1,FHOD3,FITM1,PLCXD3,TMEM65,B4GALNT3,FAM129A,CACNB2,SORBS2,GABRA4,NAV1,PPP1R12B,GJA1,CYP2J2,TNNI1,MDH1,PAM,ADPRHL1,ACAT1,MLF1,PDLIM5,HHATL,DPY19L2,ANO5,RBM20,C10orf71,USP28,SLC25A4,ASB2,PYGB,RBPMS2,BVES,HRC,SLC4A3,LIN9,FBXO32,LPL,TRIM63,CDH2,LAMA2,GPNMB,PALLD,MYO18B,TCAP,CHRM2,FGF18,MYH7B,NDUFA1,RCSD1,COX5A,ADAM23,ATP2A2,DMD,PRKAA2,PGAM2,ANLN,ABCA9,CDC42EP3,XPO4,LBH,PDK1,FBN2,USP2,SLC41A1,MYL9,RHOQ,BCO2,RIMKLA,SRPX,DCUN1D2,SCN5A,NMRK2,CT47B1,PAIP2B,PLEKHA7,HECW2,FBXL7,CLGN,STK38L,ADRB2,CA14,CAP2,MITF,HSPB1,PPARGC1A,PLCB4,CNOT11,ABHD18,SVIL,HAND2,GMPR,PPP1R1A,AFAP1L1,MPC1,ATP2B4,MFGE8,GOT1,KIDINS220,ABLIM1,SFRP1,STRN,ALPK3,RPL3L,PCDH7,CORO6,OR51B6,BZW2,PFKM,MEF2A,PRSS45,SORBS1,FASTKD1,KCNJ2,RCAN2,SAMD4A,KCNJ8,GPAT3,TRIM55,PODN,CYCS,KBTBD12,FAM189A2,ISOC1,PPM1K,QKI,COX6A2,DECR1,GBAS,SMYD2,AKAP13,LRRFIP2,AFG3L2,ACO2,SLC25A12,LARGE,USP13,ZNF436,ANKRD18A,ABCB4,CLIC4,LRRC53,RNF150,TMEM159,LTBP1,PPIC,TACC2,TGFB1I1,ADCY5,LRR1,MYBL1,CHCHD10,CRYBG3,TSPAN9,A4GALT,HSPB3,PRSS42,RRAD,MGST3,C15orf41,PLPP7,ALDOA,CPT1B,DENND5A,LRRC14B,PTP4A3,GAB1
MY	PLN,TECRL,FGF12,CSRP3,MB,ALPK2,NPPA,ANKRD1,FBXO40,MYL2,MYH7,MYLK3,MYL3,FHL2,CTNNA3,MYH6,POPDC2,TNNI3,TNNT2,NEBL,TNNC1,MYL4,LRRC10,SLC27A6,CACNA1C,SH3BGR,TBX20,HEY2,RBM24,SMYD1,FABP3,CCDC141,MYL7,MYBPC3,MLIP,ASB11,HSPB7,CRYAB,SPHKAP,CKMT2,CORIN,UNC45B,XIRP1,FSD2,RYR2,STRIP2,ALDOC,MTUS2,FILIP1,PKP2,ITGB1BP2,PFKP,NEXN,HACD1,FHOD3,PLCXD3,TMEM65,B4GALNT3,SORBS2,NAV1,PPP1R12B,GJA1,TNNI1,MDH

OCD	1,ADPRHL1,MLF1,PDLIM5,ANO5,RBM20,C10orf71,USP28,SLC25A4,ASB2,PYGB,RBPMS2,BVES,HRC,SLC4A3,LIN9,FBXO32,MYO18B,CHRM2,FGF18,MYH7B,PGAM2,ANLN,XPO4,LBH,USP2,MYL9,RHOQ,SRPX,SCN5A,PAIP2B,PLEKHA7,CLGN,STK38L,OIT3,ADRB2,CA14,CNOT11,HAND2,ATP2B4,MFGE8,GOT1,SYTL5,ABLIM1,STRN,ALPK3,CORO6,PFKM,MEF2A,KCNJ2,TRIM55,RGS5,PODN,CYCS,FAM189A2,LARGE,ABCB4,CLIC4,TMEM159,LTBP1,PPIC,TACC2,TGFB1I1,ADCY5,LRR1,MYBL1,CHCHD10,ADAL,RRAD,C15orf41,CPT1B,DENND5A,PTP4A3,GAB1
GJA1	SMPX,TMEM71,PLN,TECRL,NRAP,FGF12,CSRP3,MB,ALPK2,GPR22,NPPA,ANKRD1,FBXO40,MYL2,MYH7,MYLK3,MYL3,FHL2,POPDC3,CTNNA3,MYH6,POPDC2,TNNI3,TNNT2,NEBL,ACTC1,MPC1L,LMOD3,TNNC1,MYL4,MYOM1,LRRC10,SLC27A6,CACNA1C,SH3BGR,TBX20,CASQ2,MYOZ2,HEY2,ACTN2,RBM24,SMYD1,FABP3,CCDC141,MYL7,MYBPC3,LRRC2,MLIP,ASB11,HSPB7,SLC8A1,CRYAB,SPHKAP,CKMT2,LDB3,CORIN,UNC45B,XIRP1,FSD2,RYR2,STRIP2,ALDOC,MTUS2,TMOD1,COX7A1,FILIP1,GPRIN3,KLHL38,PKP2,MYOCD,ITGB1BP2,PFKP,NEXN,GK2,HACD1,FHOD3,FITM1,PLCXD3,TMEM65,B4GALNT3,FAM129A,CACNB2,SORBS2,GABRA4,NAV1,PPP1R12B,TNNI1,MDH1,PAM,ADPRHL1,ACAT1,MLF1,PDLIM5,HHATL,DPY19L2,ANO5,RBM20,C10orf71,USP28,SLC25A4,ASB2,PYGB,RBPMS2,BVES,HRC,SLC4A3,LIN9,FBXO32,TRIM63,LAMA2,GPNMB,PALLD,MYO18B,CHRM2,FGF18,MYH7B,NDUFA1,COX5A,ADAM23,ATP2A2,DMD,PGAM2,ANLN,ABCA9,CDC42EP3,XPO4,LBH,PDK1,FBN2,USP2,SLC41A1,MYL9,RHOQ,BCO2,SRPX,DCUN1D2,SCN5A,NMRK2,CT47B1,PAIP2B,PLEKHA7,FBXL7,CLGN,STK38L,ADRB2,CA14,CAP2,MITF,HSPB1,PPARGC1A,CNOT11,ABHD18,HAND2,GMPR,PPP1R1A,MPC1,ATP2B4,MFGE8,GOT1,ABLIM1,LAPTM4B,STRN,ALPK3,PCDH7,CORO6,OR51B6,BZW2,PFKM,MEF2A,KCNJ2,ETFDH,TRIM55,RGS5,PODN,CYCS,KBTBD12,FAM189A2,ISOC1,COX6A2,DECR1,GBAS,SMYD2,AKAP13,LRRFIP2,AFG3L2,ACO2,SLC25A12,LARGE,USP13,ZNF436,ABCB4,CLIC4,LRRC53,RNF150,TMEM159,LTBP1,PPIC,TACC2,TGFB1I1,ADCY5,LRR1,MYBL1,CHCHD10,CRYBG3,TSPAN9,A4GALT,PRSS42,RRAD,MGST3,C15orf41,PLPP7,ALDOA,CPT1B,DENND5A,LRRC14B,PTP4A3,GAB1
ATP2A2	SMPX,TMEM71,PLN,TECRL,NRAP,FGF12,CSRP3,MB,ALPK2,GPR22,NPPA,ANKRD1,FBXO40,MYL2,MYH7,MYLK3,MYL3,FHL2,POPDC3,CTNNA3,MYH6,POPDC2,TNNI3,TNNT2,NEBL,ACTC1,MPC1L,LMOD3,TNNC1,MYL4,MYOM1,LRRC10,SLC27A6,CACNA1C,LRRC39,SH3BGR,TBX20,CASQ2,MYOZ2,HEY2,ACTN2,RBM24,SMYD1,FABP3,CCDC141,MYL7,MYBPC3,LRRC2,MLIP,ASB11,HSPB7,SLC8A1,CRYAB,SPHKAP,CKMT2,LDB3,CORIN,UNC45B,XIRP1,FSD2,RYR2,STRIP2,ALDOC,MTUS2,TMOD1,COX7A1,FILIP1,GPRIN3,KLHL38,LMOD2,PKP2,ITGB1BP2,PFKP,NEXN,GK2,AKAP6,HACD1,FHOD3,FITM1,PLCXD3,TMEM65,B4GALNT3,FAM129A,CACNB2,SORBS2,GABRA4,NAV1,PPP1R12B,GJA1,CYP2J2,TNNI1,MDH1,PAM,ADPRHL1,ACAT1,MLF1,PDLIM5,HHATL,DPY19L2,ANO5,RBM20,C10orf71,USP28,SLC25A4,ASB2,PYGB,RBPMS2,BVES,HRC,SLC4A3,LIN9,FBXO32,TRIM63,CDH2,LAMA2,GPNMB,PALLD,MYO18B,TCAP,CHRM2,FGF18,MYH7B,NDUFA1,COX5A,ADAM23,DMD,PRKAA2,PGAM2,ANLN,ABCA9,CDC42EP3,XPO4,LBH,PDK1,FBN2,USP2,MYL9,RHOQ,BCO2,SRPX,DCUN1D2,SCN5A,NMRK2,CT47B1,PAIP2B,PLEKHA7,FBXL7,CLGN,STK38L,FH,ADRB2,CAP2,HSPB1,PPARGC1A,CNOT11,ABHD18,SVIL,HAND2,GMPR,PPP1R1A,AFAP1L1,MPC1,ATP2B4,MFGE8,GOT1,ABLIM1,STRN,ALPK3,RPL3L,CORO6,BZW2,PFKM,MEF2A,SORBS1,FASTKD1,KCNJ2,SAMD4A,UGP2,TRIM55,PRNP,CYCS,KBTBD12,ISOC1,QKI,COX6A2,GBAS,SMYD2,NDUFA6,AKAP13,LRRFIP2,AFG3L2,ACO2,SLC25A12,USP13,ZNF436,ABCB4,RNF150,TMEM159,LTBP1,TACC2,LRR1,MYBL1,CHCHD10,TSPAN9,A4GALT,ADAL,PRSS42,RRAD,MGST3,C15orf41,PLPP7,ALDOA,CPT1B,DENND5A,EIF1B,LRRC14B,PTP4A3
FBN2	PLN,TECRL,NRAP,FGF12,CSRP3,MB,ALPK2,NPPA,ANKRD1,FBXO40,MYL2,MYH7,MYLK3,MYL3,FHL2,POPDC3,CTNNA3,MYH6,POPDC2,TNNI3,TNNT2,NEBL,ACTC1,MPC1L,LMOD3,TNNC1,MYL4,MYOM1,LRRC10,SLC27A6,CACNA1C,SH3BGR,TBX20,CASQ2,MYOZ2,HEY2,ACTN2,RBM24,SMYD1,FABP3,CCDC141,MYL7,MYBPC3,LRRC2,MLIP,ASB11,HSPB7,SLC8A1,CRYAB,SPHKAP,CKMT2,LDB3,CORIN,UNC45B,XIRP1,FSD2,RYR2,STRIP2,ALDOC,MTUS2,TMOD1,COX7A1,FILIP1,KLHL38,PKP2,ITGB1BP2,PFKP,NEXN,HACD1,FHOD3,FITM1,PLCXD3,TMEM65,B4GALNT3,FAM129A,CACNB2,SORBS2,GABRA4,PPP1R12B,GJA1,TNNI1,MDH1,PAM,ADPRHL1,ACAT1,MLF1,PDLIM5,HHATL,DPY19L2,ANO5,RBM20,C10orf71,USP28,SLC25A4,ASB2,PYGB,RBPMS2,BVES,HRC,SLC4A3,LIN9,FBXO32,TRIM63,CDH2,LAMA2,GPNMB,PALLD,MYO18B,CHRM2,FGF18,MYH7B,NDUFA1,ATP2A2,PRKAA2,PGAM2,ANLN,ABCA9,CDC42EP3,XPO4,LBH,PDK1,USP2,SLC41A1,MYL9,RHOQ,BCO2,SRPX,DCUN1D2,SCN5A,NMRK2,CT47B1,PAIP2B,PLEKHA7,FBXL7,CLGN,ADRB2,MITF,HSPB1,PPARGC1A,CNOT

	11,ABHD18,HAND2,GMPR,AFAP1L1,MPC1,ATP2B4,MFGE8,GOT1,ABLIM1,LAPTM4B,SFRP1,STRN,ALPK3,CORO6,PFKM,MEF2A,KCNJ2,ETFDH,KCNJ8,TRIM55,QKI,DECR1,GBAS,SMYD2,NDUFA6,AKAP13,AFG3L2,ACO2,SLC25A12,LARGE,USP13,TBC1D4,ABCB4,CLIC4,RNF150,LTBP1,PPIC,TACC2,ADCY5,LRR1,MYBL1,CHCHD10,CRYBG3,TSPAN9,A4GALT,PRSS42,RRAD,MGST3,C15orf41,PLPP7,ALDOA,CPT1B,DENND5A,LRRC14B,PTP4A3
SRPX	SMPX,TMEM71,PLN,TECRL,NRAP,FGF12,CSRP3,MB,ALPK2,NPPA,ANKRD1,FBXO40,MYL2,MYH7,MYLK3,MYL3,FHL2,POPDC3,CTNNA3,MYH6,POPDC2,TNNI3,TNNT2,NEBL,ACTC1,MPC1L,LMOD3,TNNC1,MYL4,MYOM1,LRRC10,SLC27A6,CACNA1C,LRRC39,SH3BGR,TBX20,CASQ2,MYOZ2,HEY2,ACTN2,RBM24,SMYD1,FABP3,CCDC141,MYL7,MYBPC3,LRRC2,MLIP,ASB11,HSPB7,SLC8A1,CRYAB,SPHKAP,CKMT2,LDB3,CORIN,UNC45B,XIRP1,FSD2,RYR2,STRIP2,ALDOC,MTUS2,TMOD1,COX7A1,FILIP1,KLHL38,LMOD2,PKP2,MYOCD,ITGB1BP2,PFKP,NEXN,GK2,HACD1,FHOD3,FITM1,PLCXD3,TMEM65,B4GALNT3,FAM129A,CACNB2,SORBS2,GABRA4,NAV1,PPP1R12B,GJA1,TNNI1,MDH1,PAM,ADPRHL1,ACAT1,MLF1,PDLIM5,HHATL,ANO5,RBM20,C10orf71,USP28,SLC25A4,ASB2,PYGB,RBPMS2,BVES,HRC,SLC4A3,LIN9,FBXO32,TRIM63,LAMA2,GPNMB,PALLD,MYO18B,TCAP,CHRM2,FGF18,MYH7B,NDUFA1,RCSD1,COX5A,ADAM23,ATP2A2,DMD,PGAM2,ANLN,ABCA9,CDC42EP3,XPO4,LBH,FBN2,USP2,MYL9,RHOQ,BCO2,DCUN1D2,SCN5A,NMRK2,PRSS35,CT47B1,PAIP2B,PLEKHA7,FBXL7,CLGN,STK38L,ADRB2,CAP2,HSPB1,PPARGC1A,CNOT11,ABHD18,SVIL,HAND2,GMPR,AFAP1L1,MPC1,ATP2B4,MFGE8,PYGL,GOT1,ABLIM1,STRN,ALPK3,PCDH7,CORO6,OR51B6,BZW2,PFKM,LMO7,MEF2A,FASTKD1,KCNJ2,SAMD4A,KCNJ8,TRIM55,RGS5,PODN,KBTBD12,FAM189A2,QKI,COX6A2,GBAS,SMYD2,AKAP13,LRRFIP2,SLC25A12,LARGE,USP13,ZNF436,ANKRD18A,ABCB4,CLIC4,TMEM159,LTBP1,PPIC,TACC2,TGFB1I1,LRR1,MYBL1,PPP1R3C,CHCHD10,CRYBG3,TSPAN9,PRSS42,RRAD,MGST3,C15orf41,PLPP7,PMP22,ALDOA,CPT1B,DENND5A,LRRC14B,PTP4A3,GAB1
SCN5A	TMEM71,PLN,TECRL,NRAP,FGF12,CSRP3,MB,ALPK2,GPR22,NPPA,ANKRD1,FBXO40,MYL2,RD3L,MYH7,MYLK3,MYL3,FHL2,POPDC3,CTNNA3,MYH6,POPDC2,TNNI3,TNNT2,NEBL,ACTC1,MPC1L,LMOD3,TNNC1,MYL4,MYOM1,LRRC10,SLC27A6,CACNA1C,LRRC39,SH3BGR,TBX20,CASQ2,MYOZ2,HEY2,ACTN2,RBM24,SMYD1,FABP3,CCDC141,MYL7,MYBPC3,LRRC2,MLIP,ASB11,HSPB7,SLC8A1,CRYAB,SPHKAP,CKMT2,LDB3,CORIN,PRG4,UNC45B,XIRP1,FSD2,RYR2,STRIP2,ALDOC,MTUS2,TMOD1,COX7A1,FILIP1,GPRIN3,KLHL38,LMOD2,PKP2,MYOCD,ITGB1BP2,PFKP,NEXN,GK2,HACD1,FHOD3,FITM1,PLCXD3,TMEM65,B4GALNT3,FAM129A,CACNB2,SORBS2,GABRA4,NAV1,PPP1R12B,GJA1,CYP2J2,TNNI1,MDH1,PAM,ADPRHL1,MLF1,PDLIM5,HHATL,DPY19L2,ANO5,RBM20,C10orf71,USP28,SLC25A4,ASB2,PYGB,RBPMS2,BVES,HRC,SLC4A3,LIN9,FBXO32,TRIM63,LAMA2,GPNMB,PALLD,MYO18B,TCAP,CHRM2,FGF18,MYH7B,COX5A,ADAM23,ATP2A2,DMD,PRKAA2,PGAM2,ANLN,CDC42EP3,XPO4,LBH,FBN2,USP2,MYL9,RHOQ,BCO2,RIMKLA,SRPX,DCUN1D2,NMRK2,CT47B1,PAIP2B,PLEKHA7,FBXL7,CLGN,STK38L,OIT3,ADRB2,CA14,CAP2,MITF,HSPB1,PPARGC1A,CNOT11,ABHD18,SVIL,HAND2,GMPR,PPP1R1A,AFAP1L1,MPC1,ATP2B4,MFGE8,GOT1,ABLIM1,STRN,ALPK3,RPL3L,PCDH7,CORO6,OR51B6,BZW2,PFKM,MEF2A,PRSS45,SORBS1,KCNJ2,RCAN2,SAMD4A,KCNJ8,GPAT3,TRIM55,PODN,CYCS,KBTBD12,FAM189A2,QKI,COX6A2,GBAS,SMYD2,AKAP13,LRRFIP2,ACO2,SLC25A12,LARGE,ANKRD18A,ABCB4,CLIC4,LRRC53,TMEM159,LTBP1,PPIC,TACC2,TGFB1I1,ADCY5,LRR1,MYBL1,CHCHD10,CRYBG3,TSPAN9,A4GALT,HSPB3,PRSS42,RRAD,MGST3,C15orf41,PLPP7,ALDOA,CPT1B,DENND5A,LRRC14B,PTP4A3,GAB1
TBX5	ARHGAP31,GJA5
H	SMPX,TMEM71,PLN,TECRL,NRAP,FGF12,CSRP3,MB,ALPK2,GPR22,NPPA,ANKRD1,FBXO40,MYL2,MYH7,MYLK3,MYL3,FHL2,POPDC3,CTNNA3,MYH6,POPDC2,TNNI3,TNNT2,NEBL,ACTC1,MPC1L,LMOD3,TNNC1,MYL4,MYOM1,LRRC10,SLC27A6,CACNA1C,SH3BGR,TBX20,CASQ2,MYOZ2,HEY2,ACTN2,RBM24,SMYD1,FABP3,CCDC141,MYL7,MYBPC3,LRRC2,MLI

AND2	P,ASB11,HSPB7,SLC8A1,CRYAB,SPHKAP,CKMT2,LDB3,CORIN,UNC45B,XIRP1,FSD2,RYR2,STRIP2,ALDOC,MTUS2,TMOD1,COX7A1,FILIP1,GPRIN3,KLHL38,PKP2,MYOCD,ITGB1BP2,PFKP,NEXN,GK2,HACD1,FHOD3,FITM1,PLCXD3,TMEM65,B4GALNT3,FAM129A,CACNB2,SORBS2,GABRA4,NAV1,PPP1R12B,GJA1,TNNI1,MDH1,PAM,ADPRHL1,MLF1,PDLIM5,HHATL,DPY19L2,ANO5,RBM20,C10orf71,USP28,SLC25A4,ASB2,PYGB,RBPMS2,BVES,HRC,SLC4A3,LIN9,FBXO32,TRIM63,CDH2,LAMA2,GPNMB,PALLD,MYO18B,CHRM2,FGF18,MYH7B,RCSD1,COX5A,ADAM23,ATP2A2,PGAM2,ANLN,CDC42EP3,XPO4,LBH,PDK1,FBN2,USP2,MYL9,RHOQ,BCO2,SRPX,SCN5A,NMRK2,CT47B1,PAIP2B,PLEKHA7,FBXL7,CLGN,STK38L,OIT3,ADRB2,CA14,CAP2,MITF,PPARGC1A,PLCB4,CNOT11,ABHD18,SVIL,GMPR,PPP1R1A,MPC1,ATP2B4,MFGE8,GOT1,SYTL5,ABLIM1,SFRP1,STRN,ALPK3,CORO6,OR51B6,BZW2,PFKM,MEF2A,KCNJ2,RCAN2,SAMD4A,TRIM55,RGS5,PODN,CYCS,KBTBD12,FAM189A2,ISOC1,COX6A2,GBAS,SMYD2,LRRFIP2,AFG3L2,ACO2,SLC25A12,LARGE,ABCB4,CLIC4,LRRC53,RNF150,TMEM159,LTBP1,PPIC,TACC2,TGFB1I1,ADCY5,LRR1,MYBL1,CHCHD10,TSPAN9,A4GALT,PRSS42,RRAD,MGST3,C15orf41,PLPP7,ALDOA,CPT1B,DENND5A,LRRC14B,PTP4A3,GAB1
KCNJ2	SMPX,TMEM71,PLN,TECRL,NRAP,FGF12,CSRP3,MB,ALPK2,GPR22,NPPA,ANKRD1,FBXO40,MYL2,RD3L,MYH7,MYLK3,MYL3,FHL2,POPDC3,CTNNA3,MYH6,POPDC2,TNNI3,TNNT2,NEBL,ACTC1,MPC1L,LMOD3,TNNC1,MYL4,MYOM1,LRRC10,SLC27A6,CACNA1C,LRRC39,SH3BGR,TBX20,CASQ2,MYOZ2,HEY2,ACTN2,RBM24,SMYD1,FABP3,CCDC141,MYL7,MYBPC3,LRRC2,MLIP,ASB11,HSPB7,SLC8A1,CRYAB,SPHKAP,CKMT2,LDB3,CORIN,UNC45B,XIRP1,FSD2,RYR2,STRIP2,ALDOC,MTUS2,TMOD1,COX7A1,FILIP1,GPRIN3,KLHL38,LMOD2,PKP2,MYOCD,ITGB1BP2,PFKP,NEXN,GK2,HACD1,FHOD3,FITM1,PLCXD3,TMEM65,B4GALNT3,FAM129A,CACNB2,SORBS2,GABRA4,NAV1,PPP1R12B,GJA1,CYP2J2,TNNI1,MDH1,ADPRHL1,MLF1,PDLIM5,HHATL,DPY19L2,ANO5,RBM20,C10orf71,USP28,SLC25A4,ASB2,PYGB,RBPMS2,BVES,HRC,SLC4A3,LIN9,FBXO32,TRIM63,LAMA2,GPNMB,PALLD,MYO18B,TCAP,CHRM2,FGF18,MYH7B,COX5A,ADAM23,ATP2A2,DMD,PRKAA2,PGAM2,ANLN,ABCA9,CDC42EP3,XPO4,LBH,FBN2,USP2,SLC41A1,MYL9,RHOQ,SRPX,SCN5A,NMRK2,CT47B1,PAIP2B,PLEKHA7,FBXL7,CLGN,STK38L,OIT3,ADRB2,CA14,CAP2,MITF,HSPB1,PPARGC1A,CNOT11,ABHD18,SVIL,HAND2,GMPR,PPP1R1A,AFAP1L1,MPC1,ATP2B4,MFGE8,GOT1,ABLIM1,STRN,ALPK3,RPL3L,PCDH7,CORO6,OR51B6,BZW2,PFKM,MEF2A,PRSS45,RCAN2,SAMD4A,KCNJ8,TRIM55,PODN,CYCS,KBTBD12,FAM189A2,QKI,COX6A2,GBAS,SMYD2,AKAP13,LRRFIP2,SLC25A12,LARGE,ANKRD18A,ABCB4,CLIC4,LRRC53,TMEM159,LTBP1,PPIC,TACC2,TGFB1I1,ADCY5,LRR1,MYBL1,CHCHD10,CRYBG3,TSPAN9,A4GALT,HSPB3,PRSS42,RRAD,MGST3,C15orf41,PLPP7,ALDOA,CPT1B,DENND5A,LRRC14B,PTP4A3,GAB1
